# Molecular Characterization of Rotavirus C from Rescued Sloth Bears, India: Evidence of Zooanthroponotic Transmission

**DOI:** 10.3390/pathogens12070934

**Published:** 2023-07-13

**Authors:** Yashpal Singh Malik, Mohd Ikram Ansari, Mathesh Karikalan, Shubhankar Sircar, Ilayaraja Selvaraj, Souvik Ghosh, Kalpana Singh

**Affiliations:** 1 ICAR—Indian Veterinary Research Institute, Bareilly 243122, India; mikram@iul.ac.in (M.I.A.); shubhankar.sircar@wsu.edu (S.S.); 2 College of Animal Biotechnology, Guru Angad Dev Veterinary and Animal Sciences University, Ludhiana 141004, India; kalpana.iiita@gmail.com; 3 Department of Biosciences, Integral University, Lucknow 226026, India; 4 Centre for Wildlife Conservation Management and Disease Surveillance, ICAR—Indian Veterinary Research Institute, Bareilly 243122, India; mathesh.karikalan@icar.gov.in; 5 Department of Animal Sciences, Washington State University, Pullman, WA 99163, USA; 6 Agra Bear Rescue Centre, Wildlife SOS, Agra 282007, India; ilayaraja@wildlifesos.org; 7 Department of Biomedical Sciences, Ross University School of Veterinary Medicine, Basseterre P.O. Box 334, Saint Kitts and Nevis; sghosh@rossu.edu

**Keywords:** rotavirus C, sloth bear, sequence analysis, real-time phylogenetic analysis, selection pressure analysis, structure prediction analysis, NSP4, VP7, VP6 gene

## Abstract

The present study reports the detection and molecular characterisation of rotavirus C (RVC) in sloth bears (Melursus ursinus) rescued from urban areas in India. Based on an RVC VP6 gene-targeted diagnostic RT-PCR assay, 48.3% (42/87) of sloth bears tested positive for RVC infection. The VP6, VP7, and NSP4 genes of three sloth bear RVC isolates (UP-SB19, 21, and 37) were further analysed. The VP6 genes of RVC UP-SB21 and 37 isolates were only 37% identical. The sequence identity, TM-score from structure alignment, and selection pressure (dN/dS) of VP6 UP-SB37 with pig and human RVCs isolates were (99.67%, 0.97, and 1.718) and (99.01%, 0.93, and 0.0340), respectively. However, VP6 UP-SB21 has an identity, TM-score, and dN/dS of (84.38%, 1.0, and 0.0648) and (99.63%, 1.0, and 3.7696) with human and pig RVC isolates, respectively. The VP7 genes from UP-SB19 and 37 RVC isolates were 79.98% identical and shared identity, TM-score, and dN/dS of 88.4%, 0.76, and 5.3210, along with 77.98%, 0.77, and 4.7483 with pig and human RVC isolates, respectively. The NSP4 gene of UP-SB37 RVC isolates has an identity, TM-score, and dN/dS of 98.95%, 0.76, and 0.2907, along with 83.12%, 0.34, and 0.2133 with pig and human RVC isolates, respectively. Phylogenetic analysis of the nucleotide sequences of the sloth bear RVC isolates assigned the isolate UP-SB37 to genotype G12, I2 for RVC structural genes VP7 and VP6, and E1 for NSP4 genes, respectively, while isolates UP-SB19 and UP-SB21 were classified as genotype G13 and GI7 based on the structural gene VP7, respectively. The study suggests that the RVCs circulating in the Indian sloth bear population are highly divergent and might have originated from pigs or humans, and further investigation focusing on the whole genome sequencing of the sloth bear RVC isolate may shed light on the virus origin and evolution.

## 1. Introduction

Rotaviruses (RVs) are a major public health concern due to their ability to cause severe gastroenteritis in both humans and animals [1]. The viruses are non-enveloped and have a genome of 11 segments that code for six structural proteins (VP1-4, VP6, and VP7) and five or six non-structural proteins (NSP1-NSP5/NSP6) [2]. There are 11 distinct types of rotaviruses, named RVA—RVD, RVF—RVJ, that are characterised based on the inner capsid VP6 protein [3], and two putative new types, K and L, have recently been identified [4,5]. However, it is important to consider recent research findings that provide further insights into the classification of rotavirus species. Notably, a study by Johne and colleagues presents compelling evidence for the existence of species L RVs, which is the newest species to be described [5]. This study offers strong evidence supporting the classification of species L RVs. RVA, RVB, and RVC are found in mammals, including humans, swine, and cattle [6,7,8]. In humans, rotavirus infections predominantly affect children under the age of five, resulting in over 200,000 fatalities annually and accounting for 40% of hospital admissions due to diarrhea [9]. Domesticated animals such as cattle, pigs, horses, and small ruminants are also prone to enteric diseases associated with rotaviruses, causing significant mortality and economic losses through the decline in production [10,11,12].

Rotavirus C (RVC) is a significant cause of diarrhea globally, affecting individuals of all age groups [13,14,15]. Its first report dates back to 1980 when it was identified in pigs in the United States [16], and subsequent studies have identified it in humans, dairy cattle, ferrets, and dogs [17,18,19,20,21]. RVC infections in pigs have been reported worldwide, often associated with epidemics of diarrhea [22,23,24,25]. Numerous studies worldwide have linked diarrheal episodes to rotavirus C [15,26,27,28]. Moreover, genetic reassortment events between porcine RVC and bovine/human RVC have been reported [29,30], indicating the occurrence of interspecies transmission events between animals and humans [31].

Sloth bears (Melursus ursinus) are native to India, Sri Lanka, Nepal, Bhutan, and Bangladesh, with their habitat spanning from the lower regions of the Himalayas to the southern tip of the Western Ghats in India. The Indian sloth bear is listed as a Schedule I (endangered) species under the Indian Wildlife Protection Act, 1972, due to the serious threat posed to its survival in the wild as a result of poaching and habitat loss. The International Union for Conservation of Nature (IUCN) also classifies the Indian sloth bear as at risk on its Red List of Threatened Species [32]. Despite the importance of sloth bears in Indian wildlife conservation, the prevalence of RVC infection remains unknown. Furthermore, there is limited information available regarding the zoonotic potential and economic impact of RVC in sloth bears, as well as limited data on RVC detection in India, with only a few studies reporting RVC infections in humans and pigs. However, evidence of RVC infections, cross-species transmission, and reassortment in various animal species, including humans, cattle, pigs, ferrets, and dogs, highlights the emerging zoonotic potential of RVC and the importance of continuous monitoring [17,18,31].

This study aimed to identify and molecularly characterise RVC in sloth bears from different rescue centres in India. Since sloth bears rescued from Kalandars (sloth bear rearing community) are often in close proximity with humans and other animals, including pigs, we hypothesised that they are at risk of RVC infection. To test this hypothesis, we used RT-PCR and sequence analysis to determine the origin and genetic association of the RVC strains found in the sloth bears with other RVC strains reported from different geographical regions globally. We also investigated the association of sloth bears RVC with published RVC sequences from other animal species. In addition, sequence alignment, protein structure prediction, structure alignment, phylogenetic analysis, and selection pressure were also analysed for VP6, VP7, and NSP4 genes from UP-SB19, 21, and 37 RVC isolates from the sloth bear host.

In this study, we aim to investigate the genetic characteristics and origins of RVC in sloth bears by focusing on the VP6, VP7, and NSP4 genes. The VP6 gene, an inner capsid protein, allows the classification of different rotavirus species, including RVC. By analysing the VP6 gene in sloth bear RVC strains, we gain valuable insights into the molecular diversity and evolutionary relationships of this strain. Additionally, the VP7 gene plays a crucial role in antigenic properties and serotype classification. Through the analysis of VP7 gene sequences in sloth bear RVC, we aim to identify specific genotypes and compare them with other known rotavirus strains. This study aims to shed light on the genetic diversity, origins, and potential transmission sources of RVC in sloth bears, contributing to our understanding of this viral pathogen and its implications for wildlife conservation and public health. Overall, our study provides insights into the potential zoonotic risks associated with RVC infections in sloth bears and highlights the importance of continued surveillance to understand the epidemiology and evolution of RVC in different animal species.

## 2. Materials and Methods

### 2.1. Sample Collection and Processing

The study collected a total of 87 faecal samples from sloth bears at various bear rescue centers in India between 2016 and 2018. The samples were obtained from the Agra sloth bear rescue center (*n* = 60), the Bengaluru bear rescue center (*n* = 8), the Agra Mathura captive center (*n* = 18), and the Etawah Safari (*n* = 1). The samples were transported at 4 °C to the laboratory and stored immediately at −20 °C until further processing. A 10% faecal suspension (*w*/*v*) was prepared using phosphate-buffered saline (pH 7.2), and clarification was achieved through centrifugation at 5000 rpm for 10 min to eliminate coarse debris. From the supernatant, 0.5 mL was used for the viral nucleic acid extraction, and the remainder was archived for future reference.

### 2.2. RNA Extraction

To extract total RNA from the 500 µL of 10% PBS suspension, the Qiazol reagent (Qiagen GmbH, Hilden, Germany) was employed following the manufacturer’s guidelines. The extracted total RNA was then dissolved in 25 µL of nuclease-free water (NFW) and assessed for both quality and quantity using the Nanodrop Spectrophotometer (ND1000, Thermo Scientific, Waltham, MA, USA).

### 2.3. RT-PCR for Diagnosis of Rotavirus C

To detect the presence of rotavirus C (RVC), a diagnostic reverse transcription polymerase chain reaction (RT-PCR) based on the VP6 gene was carried out using specific primers RVC-VP6-DF [31] and BMJ44 [33]. The primers were designed to yield an amplicon of approximately 335 base pairs, as seen in Table 1. For cDNA synthesis, 500 ng of extracted RNA was subjected to random priming reverse transcription at 37 °C using Murine Moloney leukemia virus origin reverse transcriptase (MMLV-RT) from Promega, USA. The thermocycling conditions for PCR amplification involved an initial denaturation at 95 °C for 5 min, followed by 35 cycles of 94 °C for 20 s for denaturation, annealing at 48 °C for 20 s, and extension at 72 °C for 20 s. Lastly, a final extension at 72 °C for 5 min was performed to obtain the finished product in positive samples.

### 2.4. Cloning and Sequencing

To further characterise the identified RVC strains, both structural (VP6 and VP7 genes) and non-structural (NSP4) genes were amplified using specific primers listed in Table 1. For the amplification of the VP6 gene, two different primer pairs (Table 1) were used to obtain amplicons of different sizes (1353 bp and 584 bp) from the samples that were confirmed positive for RVC using diagnostic RT-PCR [31,34]. Similarly, VP7 gene-specific primer pair and NSP4 gene-specific primer pair were employed to amplify the respective genes (Table 1). The optimised PCR thermal conditions for all the genes included an initial denaturation step at 95 °C for 5 min, followed by 35 cycles of amplification at 94 °C for 10 s, 48 °C for 25 s, and 72 °C for 45 s, with a final extension step of 72 °C for 10 min.

The amplified gene products were cloned into the pDRIVE cloning vector (Qiagen GmbH, Hilden, Germany) and sequenced using the Big Dye terminator Sanger sequencing method on an ABI 3730xl sequencer (Eurofins Genomic India Ltd., Bangalore, India). Three RVC isolates were sequenced for VP6, VP7, and/or NSP4 genes. The length of the sequences obtained for the VP6, VP7, and NSP4 genes of RVC strain UP-SB37 were 607 bp, 1088 bp, and 477 bp, respectively, while the length of VP6 and VP7 gene sequences of UP-SB21 and UP-SB19 were 1349 bp and 1055 bp, respectively. The obtained gene sequences were either partial (VP6, 607 bp, and NSP4, 477 bp) or nearly full-length (VP7, 1055 bp, and VP6, 1349 bp).

### 2.5. NCBI GenBank Submission

The full or nearly full-length nucleotide sequences of the VP6, VP7, and NSP4 genes of the RVC strains isolated from sloth bears (UP-SB37, UP-SB21, and UP-SB19) have been deposited in the NCBI GenBank database. The accession numbers for the sequences are KX353812.1 (1349 bp, VP6 UP-SB21), KX442661.1 (1088 bp, VP7 UP-SB37), KX353813.1 (1055 bp, VP7 UP-SB19), and KX442662.1 (477 bp, NSP4 UP-SB37). The partial length VP6 sequence was deposited under the accession number KX442660.1 (607 bp, VP6 UP-SB37).

### 2.6. Related Sequences Search and Phylogenetic Analysis

To gain insights into the genetic diversity and evolutionary relationships of the sloth bear RVC isolates, this study conducted the molecular characterisation of the RVC isolates found in sloth bears targeting the VP6 and VP7 genes, as well as the non-structural NSP4 gene. First BLAST analysis [35] was performed for each of the above-mentioned bear RVC gene sequences submitted to NCBI. Homologous sequences to each of the query sequences (for VP6 UP-SB37, VP6 UP-SB21, VP7 UP-SB37, and NSP4 UP-SB37 genes) were filtered based on the 95–100% query coverage with maximum identity. Then, protein sequences of all the genes were obtained from the NBCI protein database. Subsequently, multiple sequence alignment (MSA) was performed using CLUSTAL omega [36] for each of the protein sequences, along with its homologous protein sequences obtained from BLAST. Later, a relative time phylogenetic tree was prepared, using MSA for each of the proteins using the Realtime-ML algorithm on the tree. This was prepared using the Maximum likelihood algorithm and JTT matrix-based model with 1000 bootstraps using PHYLIP [37]. Finally, phylogenetic trees were visualised in MEGA11 [38].

### 2.7. Structure Prediction and Alignment

This analysis was undertaken to gain insight into the potential structural similarities and differences between these RVC proteins and their counterparts from other hosts. In addition to the phylogenetic analysis, we performed a structural analysis of the NSP4 UP-SB37, VP6 UP-SB-37, VP6 UP-SB21, VP7 UP-SB19, and VP7 UP-SB37 proteins along with the best match sequences from all other hosts found in each case (details provided in Supplementary Table S1). These protein sequences were modelled by threading modelling using I-TASSER [39], as no template with >20% sequence identity was found in the PDB protein structure database. The best model for each protein was selected based on structure assessment and then compared with the structures of the best match sequences from other hosts using TM-align [40].

### 2.8. Selection Pressure Analysis

The codon-wise and gene-wise selection pressure analysis was performed for all the genes (VP6 UP-SB37, VP6 UP-SB21, VP7 UP-SB19, VP7 UP-SB37, and NSP4 UP-SB37) of RVC isolates from various hosts using HYPHY/Datamonkey [41] using the codon-wise MSA file of nucleotide sequences obtained using MUSCLE [42]. The analysis was carried out to determine the selection pressures acting on individual codons and entire genes in terms of non-synonymous/synonymous rate ratio (dN/dS).

## 3. Results and Discussion

### 3.1. RVC Occurrence in Indian Sloth Bears

The results of the RVC VP6 gene-specific diagnostic RT-PCR assay showed that 48.3% (42/87) of the sloth bear samples were positive for RVC. It is noteworthy that the samples also tested positive for other viruses, such as Rotavirus-A (RVA) and Picobirnavirus (PBV), with RVA being identified in 24.1% (21/87) of samples and six samples testing positive for PBVs. This study is the first to report the detection of RVC in the sloth bear population, and the viral aetiology of RVC in this population has not been extensively investigated. However, RVC is known to infect a wide range of hosts, including humans and various animals such as porcine, bovines, ferrets, and dogs [43]. It is typically found in suckling and weaned piglets, and there have been reports of co-infections with other viruses [16,24]. Previous studies from India have reported the detection of RVC in human samples using RT-PCR assays [6,44]. The present study provides valuable insights into the prevalence of RVC in sloth bears and its co-infection with other viruses (Rotavirus and PBVs) in this population.

Studies on RVC prevalence in various animal species have shown wide variations, ranging from 0.3% to 46% [15,25,45]. In pigs, RVC has been detected in both diarrheic and non-diarrheic samples, with reported prevalence rates of 26.3% and 31.3%, respectively [24,46]. However, lower prevalence rates have also been reported in some studies, such as 12.0% in piglet faecal specimens from India and 4.4% in asymptomatic piglets from Ireland [22,31]. The variability in RVC prevalence rates across different studies may be attributed to factors such as the sample size, sensitivity of the detection method used, and geographical and environmental factors. To note, the current study reported a higher prevalence of RVC (48.3%) in asymptomatic sloth bears. This may be attributed to the close association of sloth bears with humans, as their habitat is located in areas with high porcine populations. It is worth noting that the detection of other viruses, such as RVA and PBV, in the sloth bear samples suggests that co-infections with multiple viruses are common in this species. The detection of RVC in sloth bears highlights the need for further studies to understand the epidemiology and potential zoonotic implications of this virus in this host species.

### 3.2. Genetic Analysis of VP6 Gene from Sloth-Bear RVC Isolates, UP-SB37, and UP-SB21

#### 3.2.1. Sequence Analysis

The analysis of the VP6 gene sequences from the sloth bear RVC isolates (UP-SB21 and UP-SB37) showed a significant divergence, with only 37% identity and 41.85% coverage with each other; therefore, separate analyses were conducted for each sequence. BLAST searches were performed using each sequence, and a total of 105 sequences (Supplementary Table S2, and the fasta file are provided in the Supplementary files folder) with a minimum sequence identity of 80.46% and 99% coverage with VP6 UP-SB37 were analysed. It matched different human RVC strains between 94.89–99.01% identity and pig RVC strains between 80.46–99.67% identity. Similarly, a sum of 122 sequences (Supplementary Table S3 and the fasta file are provided in the Supplementary files folder) with a minimum sequence identity of 82.13% and query coverage of 95% with VP6 UP-SB21 were found to match with different RVC strains from bovine (82.13–86.67% identity), human (84.38–83.10% identity), and pig (99.63–83.06% identity) hosts. In the present study, it was found that VP6 UP-SB37 and UP-SB21 genes were closer to pigs and humans than to bovines. It is reported in the previous studies as well that the RVC is more similar to human and porcine RVC and shares lower sequence identities with bovines and other animals. However, the bovine RVC from Japan was found to be less related to humans and porcine [47]. They reported 57.6–82.1% identity to humans and 56.5–82.6% to pig RVC at the nucleotide sequence level. In agreement with the present study, a report from India reported 93.9% identity of pig strain with human RVC strain [31]. These results suggest that sloth bears may be susceptible to RVC that are similar to those that infect humans and pigs, highlighting the potential for interspecies transmission of RVC.

#### 3.2.2. Genotyping and Phylogenetic Analyses

In the present study, the genetic classification of the two strains of the RVC VP6 gene was performed on the basis of a cut-off value of 87% nucleotide identity as recently described [43,48] using pairwise identity frequency graphs approved by the ICTV [49]. In the present study, the two RVC strains were classified into two VP6 genotypes. The sloth bear strain UP-SB21 clusters with the porcine genotype (I7) show a 99.63% nucleotide similarity, while the isolate UP-SB37 clusters with the human genotype (I2) have a 99.01% nucleotide identity. The two RVC strains from the sloth bear were grouped into human genotype I2 and porcine genotype I7. The recognition of RVC VP6 genotype I2 and I7 are the first incidence of detection and molecular characterisation in sloth bears in India. There are previous reports of I2 and I7 genotype detection in RVC strains from humans and pigs [6,31,45]. The sloth bear RVC was related to both human and porcine RVC.

**Figure 1:**
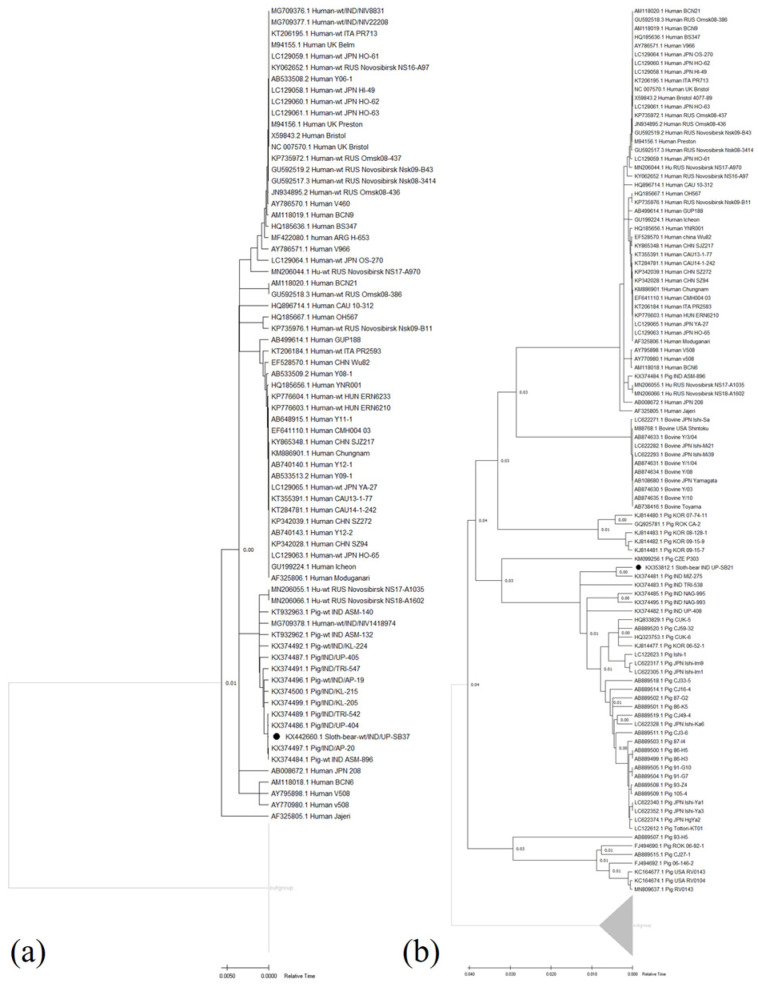
Relative time phylogenetic trees of protein sequences of VP6 gene from UP-SB37 and other strains of RVC virus in various hosts (**a**); of VP6 gene from UP-SB21 and other strains of RVC virus in various hosts (**b**). The sloth-bear RVC sequences are indicated by the dark black circle “●”. The tree was generated using Realtime-ML algorithm on the tree prepared using Maximum likelihood algorithm and JTT matrix-based model with 1000 bootstraps using PHYLIP and visualized using MEGA11 software. The bar indicates relative time scale.

The relative time-based phylogenetic trees for VP6 UP-SB37 and VP6 UP-SB21 genes, respectively, were prepared from the protein sequences of all gene sequences and shown in Figure 1a,b. The results from the phylogenetic study show that VP6 UP-SB37 was grouped with the Indian strains of porcine UP-404, AP-20, ASM-896, and TRI-547 (Figure 1a), while VP6 UP-SB21 was grouped with the Indian strain of porcine MZ-275 on the basis of the relative time of divergence (Figure 1b).

#### 3.2.3. Structure Alignment and Analysis

**Figure 2:**
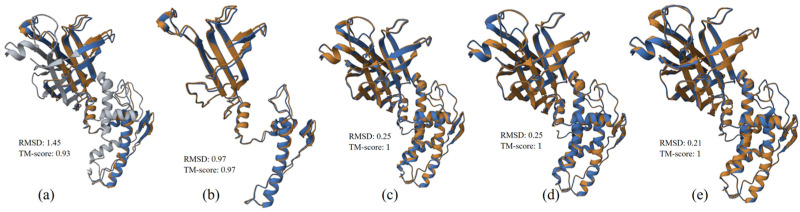
VP6 UP-SB37 protein structure (brown) aligned with proteins (blue) from human (**a**) and pig RVC isolates (**b**); VP6 UP-SB21 protein structure (brown) aligned with proteins (blue) from bovine (**c**), human (**d**), and pig RVC isolates (**e**). RMSD value indicates how well the structure alignment is, and TM score indicates the relatedness between two structures.

The structural alignments of VP6 UP-SB37 protein with VP6 protein from human and porcine (.pdb files are provided in the Supplementary files folder) hosts were performed and provided in Figure 2a,b, respectively. The results show that there was a better alignment of VP6 UP-SB37 protein with protein from the pig (TM-score 0.97 and RMSD 0.97) in comparison to human (TM-score 0.93 and RMSD 1.45) isolates. Similarly, the structural alignment of VP6 UP-SB21 protein with VP6 protein from bovine, human, and pig (.pdb files are provided in the Supplementary files folder) hosts was performed and shown in Figure 2c–e, respectively. The results from the structure alignments analysis of VP6 UP-SB21 protein also found that the alignment was better for protein from pig (TM-score 1 and RMSD 0.21) in comparison to human (TM-score 1 and RMSD 0.25) and bovine (TM-score 1 and RMSD 0.25) isolates. Yet both structure alignments for humans, bovines, and pigs show considerable relatedness in terms of TM-score (>0.7) and good alignment in terms of RMSD (<3.0) value with UP-SB37; however, it was the best for pigs.

#### 3.2.4. Selection Pressure Analysis

The results of the selection pressure analysis state that VP6 UP-SB37 and UP-SB21 genes have purifying and positive selection pressure, respectively, on them across the various strains of different hosts studied in the present study (Table 2). The results for codon-wise selection pressure analyses for VP6 UP-SB37 and VP6 UP-SB21 genes are provided in Supplementary Tables S4 and S5, respectively. It was found that none of the codons had positive selection pressure in the VP6 UP-SB37 gene, while 19 codons had negative selection pressure in the VP6 UP-SB21 gene. Species-wise selection pressure analyses were also performed (Table 2), and it was found that the VP6 UP-SB37 gene had positive selection (dN/dS = 1.718) along with pig isolates, however, had negative selection (dN/dS = 0.0340) along human isolates. Similar results were found for the VP6 UP-SB21 gene as it had positive selection (dN/dS = 3.7696) along with pig isolates and had negative selection (dN/dS = 0.0648) along with human isolates.

These results from the sequence, structural alignment, phylogenetic, and species-wise selection pressure analyses of the VP6 gene suggest that RVC in sloth bears may have been acquired from a porcine source which may be adapted by reassortment in the sloth bear population.

### 3.3. Genetic Analysis of VP7 Gene from Sloth-Bear RVC Isolates, UP-SB37, and UP-SB19

#### 3.3.1. Sequence Analysis

The VP7 gene sequences from the two sloth bear RVC isolates (UP-SB19 and UP-SB37) were determined and found to have 79.98% identity with 96% coverage with each other. Therefore, a combined analysis was performed for both, using VP7 UP-SB37 for searching similar sequences. A sum of 49 sequences (Supplementary Table S6 and the fasta file are provided in the Supplementary files folder) with a minimum sequence identity of 77.79% and a coverage of 96% with VP7 UP-SB37 were found to match with different RVC strains from human (77.79–77.98% identity) and pig hosts (77.85–88.40% identity) along with VP7 UP-SB21. In the previous studies, a higher percentage identity, up to 99%, has been reported in the porcine RVC VP7 gene with other porcine strains [44]. The RVC VP7 strain from sloth bears having a lower (88.40%) percent identity, which was the highest in this case, suggests that this strain may have undergone reassortment or was acquired from a porcine origin.

#### 3.3.2. Genotyping and Phylogenetic Analyses

**Figure 3:**
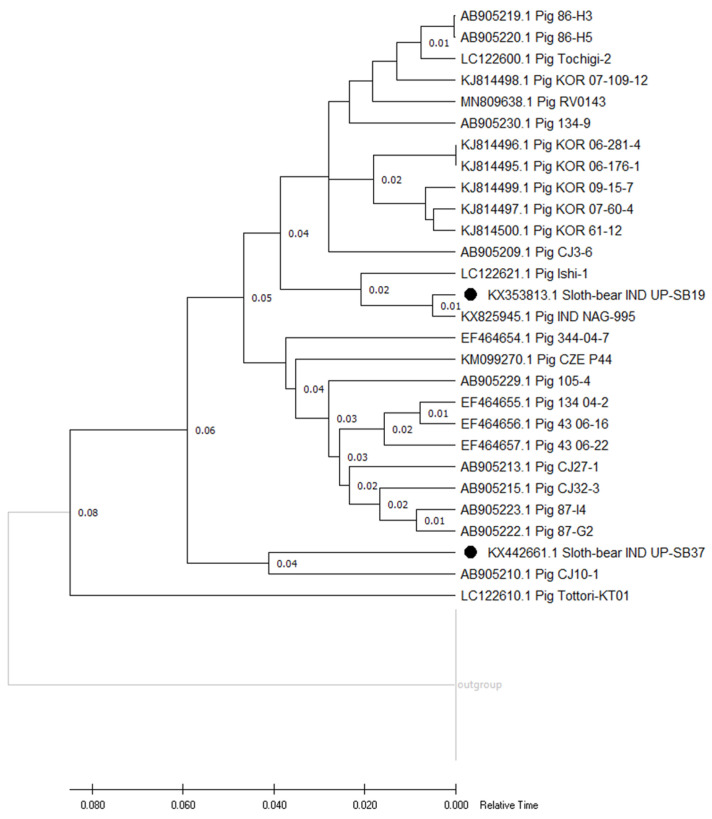
Relative time phylogenetic tree of protein sequence of VP7 gene from UP-SB37 and 19 along with other strains of RVC virus in various hosts. The present study RVC sequences are indicated by the dark black circle “●”. The tree was generated using Realtime-ML algorithm on the tree prepared using Maximum likelihood algorithm and JTT matrix-based model with 1000 bootstraps using PHYLIP and visualized using MEGA11 software. The bar indicates relative time scale.

The phylogenetic analysis of the two sloth bear isolates (UP-SB19 and UP-SB37) reveals the clustering of the isolates into two separate clusters (Figure 3). The isolate UP-SB19 clusters with the porcine Indian isolate NAG-995, while the isolate UP-SB37 cluster with the porcine isolate CJ10-1 from Japan. It was previously reported that one isolate of RVC showing 82.2–84.4% identity did not cluster with other RVC strains of the same study [50]. In another study, it was reported that the VP7 sequences from porcine RVCs can be divided into multiple clusters with cut-off values of 85% in comparison to VP7 from other species. This further supports our findings that the two isolates, UP-SB19 and UP-SB37, are more similar to porcine isolates [25]. Most strains analysed in this study shared nucleotide identities with previously reported Asian RVC strains (Indian and Japanese strains) [31,51]. Taken together, these data suggest that the same RVC genotypes are in circulation within Asian countries. The genetic classification of the RVC VP7 gene of the two isolates, UP-SB19 and UP-SB37, was performed at an 85% cut-off value as described previously [43]. The gene sequences of the two isolates show 86% identity and are grouped together in the RVC VP7 gene phylogeny. The strain UP-SB19 cluster with porcine genotype (G13), while the UP-SB37 cluster with porcine genotype (G12). The RVC VP7 genotype G12 has been reported in healthy pigs [52], as in our case, both the genotypes (G12 and G13) were found in asymptomatic sloth bears. Similarly, in the case of RVA, VP7 genotype isolates have been detected in asymptomatic animals [53,54,55]. Asymptomatic RVA infections are considered to be caused by some of the common genotype strains in pigs [56,57,58]. Though some studies suggest that such rare genotype RVAs rarely cause diarrhea, a phenotype not usually associated with RVA [59,60]. It is believed that RVCs have been developing along the same lines as RVA. To gain knowledge of the clinical symptoms and epidemiology of sloth bear G12 and G13 RVC genotype infections, further etiological and epidemiological examinations are required.

#### 3.3.3. Structure Alignment and Analysis

**Figure 4:**
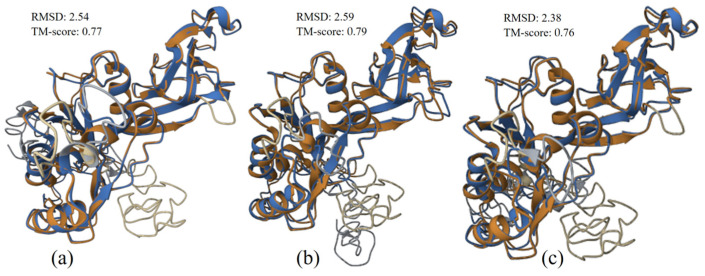
VP7 UP-SB37 protein structure (brown) aligned with proteins (blue) from human (**a**), UP-SB19 (**b**), and pig RVC isolates (**c**). RMSD value indicates how well the structure alignment is, and TM Score indicates the relatedness between two structures.

The structural alignments of VP7 UP-SB37 protein with VP7 protein from human, UP-SB21, and porcine (.pdb files are provided in Supplementary files folder) hosts were performed and provided in Figure 4a–c, respectively. The results from the structure alignment of VP7 UP-SB37 also found that the alignment with protein from porcine (TM-score 0.76 and RMSD 2.38) was better in comparison to protein from human (TM-score 0.77 and RMSD 2.54) based on RMSD value. Yet, both structure alignments with human and porcine showed considerable relatedness in terms of TM-score (>0.7) and good alignment in terms of RMSD (<3.0) value with UP-SB37 along with protein from porcine.

#### 3.3.4. Selection Pressure Analysis

The results of the selection pressure analysis of VP7 UP-SB37 and UP-SB19 genes state that both genes had positive selection pressure on them across the various strains of different hosts studied in the present study (Table 2). The results for codon-wise selection pressure analysis for VP7 UP-SB37 and VP7 UP-SB19 genes are provided in Supplementary Table S7. It was found that a sum of seven codons had negative selection pressure in the VP7 UP-SB gene. However, from species-wise selection pressure analyses (Table 2), it was found that VP7 UP-SB37 and UP-SB19 genes had more positive selection (dN/dS = 5.3210) along with porcine isolates and had less positive selection pressure (dN/dS = 4.7483) along with human isolates.

These results from the sequence, structural alignment, phylogenetic, and species-wise selection pressure analyses of the VP7 gene suggest that RVC in sloth bears may have been acquired from a porcine source which may be adapted by reassortment in the sloth bear population.

### 3.4. Genetic Analysis of NSP4 Gene from Sloth-Bear RVC Strain UP-SB37

#### 3.4.1. Sequence Analysis

The NSP4 gene sequences from the sloth bear RVC isolates (UP-SB37) were determined and were found having sequence identity with NSP4 sequences of other porcine (80.75–98.95% identity) and human (82.91–83.12% identity) RVC isolates from GenBank database (Supplementary Table S8 and the fasta file are provided in the Supplementary files folder). The UP-SB37 isolate showed the highest identity (98.95%) with the Indian porcine strain Por-993. A previous study from the Czech Republic reported a higher NSP4 gene sequence identity of 90% with the Cowden strain [13]. One of the reports from Russia reported the percent nucleotide identity of 67.7% in five porcine RVC strains and 64.4% in two RVC bovine isolates [58].

#### 3.4.2. Genotype and Phylogenetic Analyses

**Figure 5:**
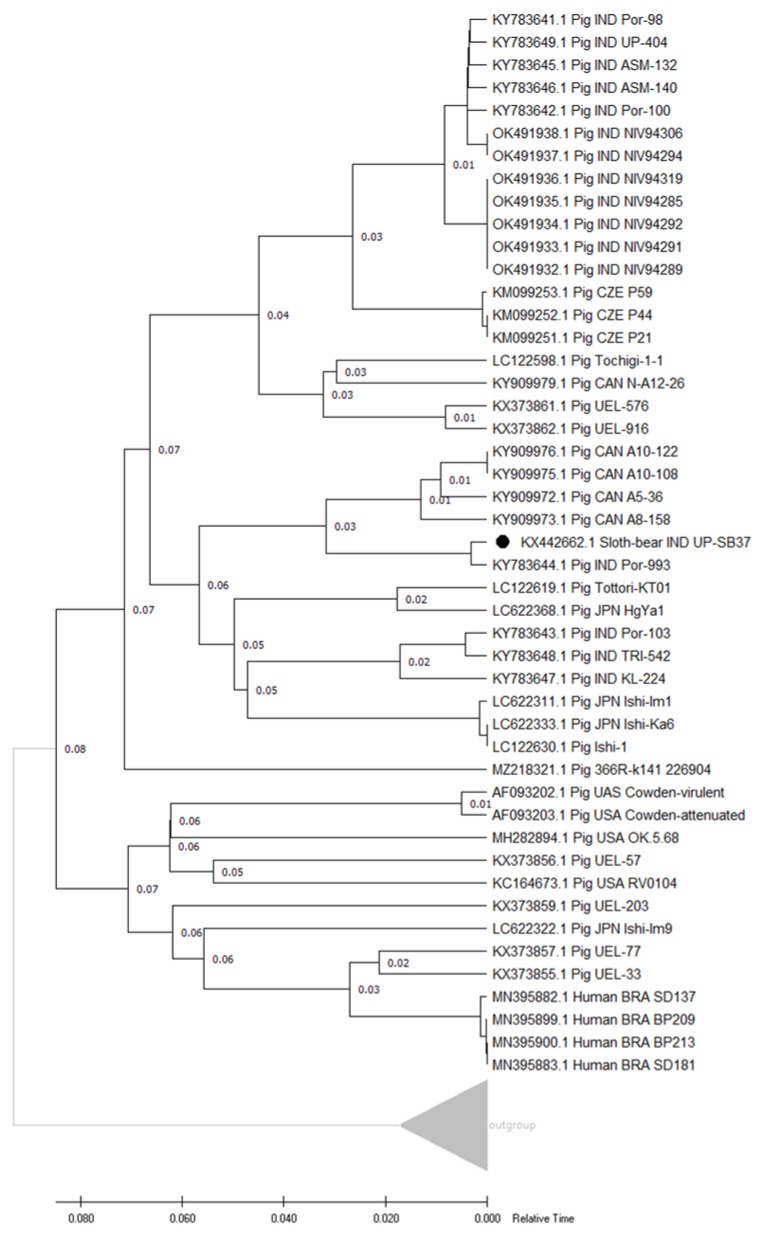
Relative time phylogenetic tree of protein sequence of NSP4 gene from UP-SB37 and other strains of RVC virus in various hosts. The present study RVC sequence is indicated by the dark black circle “●”. The tree was generated using Realtime-ML algorithm on the tree prepared using Maximum likelihood algorithm and JTT matrix-based model with 1000 bootstraps using PHYLIP and visualized using MEGA11 software. The bar indicates relative time scale.

From the results of phylogenetic analysis for the NSP4 gene, it was found that the UP-SB37 RVC isolate clustered the with Indian porcine strain Por-993 RVC strain (Figure 5). In a previous study, the genetic classification of the RVC NSP4 gene was based on an 81% cut-off value which determined five E (E1–E5) genotypes [43]. The isolate UP-SB37 showed 86% identity and was grouped with the RVC NSP4 gene with porcine genotype (E1). This may reflect the geographical segregation and the existence of regional RVC strain [58]. These data indicate that RVCs with at least two different genome constellations might be circulating in India and correlate with other studies, which showed the simultaneous circulation of RVCs of different lineages in one country [58,59,60].

#### 3.4.3. Structure Alignment and Analysis

**Figure 6:**
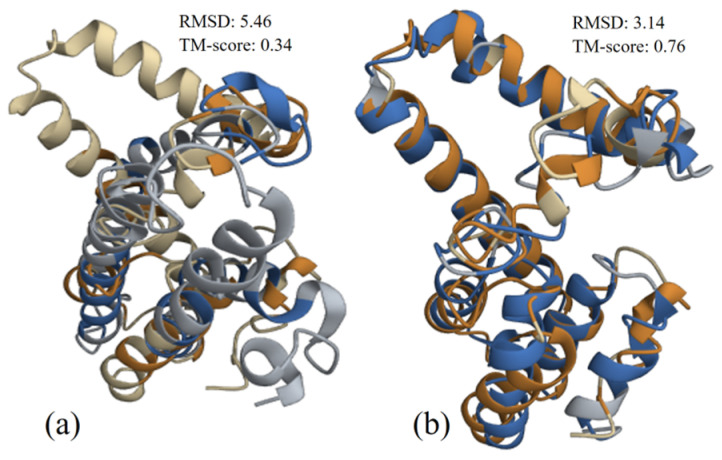
NSP4 UP-SB37 protein structure (brown) aligned with proteins (blue) from human (**a**) and pig RVC isolates (**b**). RMSD value indicates how well the structure alignment is, and TM Score indicates the relatedness between two structures.

The structure alignments of NSP4 UP-SB37 protein with the protein from human and pig (.pdb files are provided in the Supplementary files folder) isolates were performed and provided in Figure 6a,b, respectively. From the structure alignment results, it can be seen that the NSP4 protein of UP-SB37 has better alignment with porcine than with human; similarly, TM-score results suggests that it has better relatedness with porcine (TM-score 0.76) and very poor relatedness with human (TM-score 0.34).

#### 3.4.4. Selection Pressure Analysis

The results of the selection pressure analysis of the VP6 UP-SB37 gene (Table 2) show that it had purifying selection pressure on it across the strains of different hosts studied in the present study. The results for codon-wise selection pressure analysis for the NSP4 UP-SB37 gene are provided in Supplementary Table S9, and it was found that only 3 codons had positive selection pressure in the NSP4 UP-SB37 gene. However, species-wise selection pressure analyses (Table 2) reveal that the NSP4 UP-SB37 gene had less negative selection pressure (dN/dS = 0.2907) along with pig isolates and had more negative selection pressure (dN/dS = 0.2133) along with human isolates.

These results from sequence, structural alignment, phylogenetic, and species-wise selection pressure analyses of the NSP4 gene suggest that RVC in sloth bears may have acquired this gene from pigs which may be adapted by reassortment in the sloth bear population.

In conclusion, the present study is the first report on the detection and molecular characterisation of RVCs in endangered rescued sloth bear populations using RT-PCR. Our data provide additional information that RVC may remain asymptomatic in sloth bears, although they have been associated with gastroenteritis in other animals, including humans. The results from the sequence, structural alignment, phylogenetic, and species-wise and codon-wise selection pressure analyses of VP6, VP7, and NSP4 genes suggest that RVC in sloth bears may have a pig or human origin, which may adapt in sloth bears due to the close proximity of rescued sloth bears used in the present study with both human and pig populations. The present study could be helpful to the research community in understanding the probable transmission of the RVC virus from a pig or human host to a sloth bear and the favour of selection pressure in this adaptation. Further, investigations are required targeting the whole genome sequencing of the circulating sloth bear RVC isolates and their role in causing disease.

## Figures and Tables

**Table 1:** Primers used in the study for diagnosis and sequencing of rotavirus C in the samples.

Primers	Diagnostic, Genotyping, and Sequencing Primer	Annealing Temperature (°C)	Amplicon Size (bp)	References
RVC VP6-F & RVC VP6-R	5′-GGCTTTAAAAATCTCATTCA-3′	48	1353 bp	[34]
	3′-AGCCACATAGTTCACATTTC-5′			
RVC-VP6-PF & BMJ44	5′ARTCHGTTCTATGYGATTC3′	48	335	[31,33]
	3′AGCCACATAGTTCACATTTC5′			
RVC-VP6-PF & RVC-VP6-PR	5′TCATACTGGGGCATTGGAAC3′	48	584	[31]
	3′GCCAAGTGTTTGATTATTAGG5′			
GrC VP7-20F & GrC VP7-1062R	5′GCTGTCTGACAAACTGGTC3′	48	1241	[13]
	3′GCCACATGATCTTGTTTACGC5′			
RVC-NSP4-CDS-FP & RVC-NSP4-CDS-RP	5′CTCTACGAAGCAATGGAGTTCATCAA3′	48	477	[31]
	3′AGCGCAGAAGATTCATAGACA5′			

**Table 2:** Overall and species-wise selection pressure on VP6, VP7, and NSP4 genes from UP-SB19, UP-SB 21, and UP-SB 37, along with other isolates of RVC.

Genes	Significant Codon Sites (*p* ≤ 0.01)	Total Substitutions/Sites	dN/dS *	Selection Pressure	dN/dS with Porcine	dN/dS with Human
NSP4 UP-SB37	66	2.673	0.2878	Purifying selection	0.2907	0.2133
VP6 UP-SB21	179	3.192	3.6590	Positive selection	3.7696	0.0648
VP6 UP-SB37	253	2.515	0.0393	Purifying selection	1.718	0.0340
VP7 UP-SB37 & UP-SB19	79	2.291	5.4405	Positive selection	5.3210	4.7483

* dN/dS = non-synonymous/synonymous rate ratio.

## Data Availability

Additional data are available in Supplementary Tables S1–S9.

## Supplementary Materials

The following supporting information can be downloaded at: https://www.mdpi.com/article/10.3390/pathogens12070934/s1, Supplementary Table S1: Best templates used for structure prediction for VP6, VP7, and NSP4 proteins from different hosts along with results of structure prediction. Supplementary Table S2: BLAST results of VP6 UP-SB37 gene from sloth bear with accession ID, host, country, and strain details along with sequence identity and query coverage. Supplementary Table S3: BLAST results of VP6 UP-SB21 gene from sloth bear with accession ID, host, country, and strain details along with sequence identity and query coverage. Supplementary Table S4: Codon-wise selection pressure shown for VP6 UP-SB37 gene of RCV virus from sloth bear and other hosts. Supplementary Table S5: Codon-wise selection pressure shown for VP6 UP-SB21 gene of RCV virus from sloth bear and other hosts. Supplementary Table S6: BLAST results of VP7 UP-SB37 gene from sloth bear with accession ID, host, country, and strain details along with sequence identity and query coverage. Supplementary Table S7: Codon-wise selection pressure shown for VP7 gene of RCV virus from sloth bear (UP-SB37 and UP-SB19) and other hosts. Supplementary Table S8: BLAST results of NSP4 gene from sloth bear with accession ID, host, country, and strain details along with sequence identity and query coverage. Supplementary Table S9: Codon-wise selection pressure shown for NSP4 gene of RCV virus from sloth bear (UP-SB37) and other hosts. Fasta file are provided in the Supplementary files folder.
