# Supplementary material for: Molecular Characterization of Rotavirus C from Rescued Sloth Bears, India: Evidence of Zooanthroponotic Transmission
Source: Pathogens. 2023 Jul 13;12(7):934. doi: 10.3390/pathogens12070934 (PMC10384673; doi:10.3390/pathogens12070934)
Supplement: Supplementary file 1 [file pathogens-12-00934-s001.zip › Supplementary Table S1-9.pdf]

Supplementary Table S1: Best templates used for structure prediction for VP6, VP7 and NSP4 proteins from different host

Proteins	Accession ID, Host, Country, Strain	Best templates	C-score
NSP4	MN395882.1, Human, BRA, SD137	5lqxV, 1fewA, 7m6kA, 5o8kA, 4cr2A, 5j3nA, 7apkG, 1vt4A, 2h7oA	-3.84
	KY783644.1,Pig,IND,Por-993	5j3nA, 1fewA, 7m6kA, 6wlbA, 4cr2A, 7apkG, 1vt4A, 6ek4A, 2pff	-4.05
	KX442662.1,SB,IND,UP-SB37	5j3nA, 1fewA, 7m6kA, 6wlbA, 4cr2A, 7apkG, 1vt4A, 6ek4A, 2pff	-4.02
VP6	KX353812.1,Sloth-bear,IND,UP-SB21	3kz4O, 3kz4A, 7twcA, 3kz4, 1qhdA, 3kz4O, 7aqxA	-1.84
	KX442660.1,Sloth-bear,IND,UP-SB37	3kz4O, 3kz4A, 7z7hF, 3kz4, 1qhdA, 7tcrC	-1.60
	AF162434.1,Bovine,USA,WD534tc	3kz4O, 3kz4A, 7qfpA, 3kz4, 1qhdA, 7aqxA	1.84
	KT206195.1,Human,ITA,PR713	3kz4O, 3kz4A, 7qfpA, 3kz4, 1qhdA, 7acyA	1.83
	KX374497.1,Pig,IND,AP-20	3kz4O, 3kz4A, 7qfpA, 3kz4, 1qhdA, 7tcrC	1.62
VP7	KX353813.1,Sloth-bear,IND,UP-SB19	3gztQ, 3fmgA, 7fivB, 3gzt, 7rwkA	-2.05
	KX442661.1,Sloth-bear,IND,UP-SB37	3gztQ, 3fmgA, 7fivB, 3gzt, 7pe9I	-1.90
	AF225554.1,Human,T97,190	3gztQ, 3fmgA, 7fivB, 3gzt, 7rwkA	-2.05
	AB905210.1,Pig,CJ10-1	3gztQ, 3fmgA, 7fivB, 3gzt, 7rwkA	-1.94

Supplementary Table S2: BLAST results of VP6 UP-SB37 gene from sloth bear with accession ID, host, country and strain details along with sequence identity and query coverage

**S.No.**	**Accession ID, Host, Country, Strain**	**% coverage**	**% identity**
1.	KX442660.1,Sloth-bear,IND,UP-SB37	100%	100.00%
2.	KX374497.1,Pig,IND,AP-20	100%	99.67%

3.	KX374486.1,Pig,IND,UP-404	99%	99.67%
4.	KX374491.1,Pig,IND,TRI-547	99%	99.50%
5.	KX374489.1,Pig,IND,TRI-542	99%	99.50%
6.	KX374487.1,Pig,IND,UP-405	99%	99.50%
7.	KX374500.1,Pig,IND,KL-215	99%	99.50%
8.	KX374499.1,Pig,IND,KL-205	99%	99.50%
9.	KT932963.1,Pig,IND,ASM-140	100%	99.34%
10.	KX374484.1,Pig,IND,ASM-896	100%	99.18%
11.	KX374496.1,Pig,IND,AP-19	99%	99.34%
12.	KT932962.1,Pig,IND,ASM-132	100%	99.01%
13.	KT206195.1,Human,ITA,PR713	100%	99.01%
14.	MG709378.1,Human,IND,NIV1418974	100%	98.85%
15.	KX374492.1,Pig,IND,KL-224	99%	98.84%
16.	MN206055.1,Hu,RUS,NS17-A1035	100%	98.68%
17.	MG709376.1,Human,IND,NIV8831	100%	98.68%
18.	MN206066.1,Hu,RUS,NS18-A1602	100%	98.35%
19.	LC129064.1,Human,JPN,OS-270	100%	98.35%
20.	AY795898.1,Human,V508	100%	98.35%
21.	AY786571.1,Human,V966	100%	98.35%
22.	AY770980.1,Human,v508	100%	98.19%
23.	MF422080.1,human,ARG,H-653	100%	97.86%
24.	HQ185636.1,Human,BS347	100%	97.86%
25.	AM118019.1,Human,BCN9	100%	97.86%
26.	AY786570.1,Human,V460	99%	97.52%
27.	AF325805.1,Human,Jajeri	99%	97.52%
28.	MG709377.1,Human,IND,NIV22208	100%	97.36%
29.	KT206184.1,Human,ITA,PR2593	100%	97.36%
30.	EF528570.1,Human,CHN,Wu82	100%	97.36%
31.	JN934895.2,Human,RUS,Omsk08-436	100%	97.20%

32.	GU592517.3,Human,RUS,Nsk08-3414	100%	97.20%
33.	GU592519.2,Human,RUS,Nsk09-B43	100%	97.20%
34.	AB533509.2,Human,Y08-1	100%	97.20%
35.	HQ896714.1,Human,CAU 10-312	100%	97.20%
36.	HQ185667.1,Human,OH567	100%	97.20%
37.	HQ185656.1,Human,YNR001	100%	97.20%
38.	LC129063.1,Human,JPN,HO-65	100%	97.03%
39.	KP735976.1,Human,RUS,Nsk09-B11	100%	97.03%
40.	KP776604.1,Human,HUN,ERN6233	100%	97.03%
41.	KP776603.1,Human,HUN,ERN6210	100%	97.03%
42.	AB648915.1,Human,Y11-1	100%	97.03%
43.	GU199224.1,Human,Icheon	100%	97.03%
44.	EF641110.1,Human,CMH004,03	100%	97.03%
45.	AB008672.1,Human,JPN,208	100%	97.03%
46.	KY865348.1,Human,CHN,SJZ217	100%	96.87%
47.	KP735972.1,Human,RUS,Omsk08-437	100%	96.87%
48.	NC_007570.1,Human,UK,Bristol	100%	96.87%
49.	KM886901.1,Human,Chungnam	100%	96.87%
50.	AB740140.1,Human,Y12-1	100%	96.87%
51.	AB533513.2,Human,Y09-1	100%	96.87%
52.	X59843.2,Human,Bristol	100%	96.87%
53.	M94156.1,Human,UK,Preston	100%	96.87%
54.	AB499614.1,Human,GUP188	100%	96.71%
55.	MN206044.1,Hu,RUS,NS17-A970	100%	96.71%
56.	LC129065.1,Human,JPN,YA-27	100%	96.71%
57.	LC129061.1,Human,JPN,HO-63	100%	96.71%
58.	LC129060.1,Human,JPN,HO-62	100%	96.71%
59.	LC129058.1,Human,JPN,HI-49	100%	96.71%
60.	KT355391.1,Human,CAU13-1-77	100%	96.71%

61.	KT284781.1,Human,CAU14-1-242	100%	96.71%
62.	KP342039.1,Human,CHN,SZ272	100%	96.71%
63.	AB740143.1,Human,Y12-2	100%	96.71%
64.	AB533508.2,Human,Y06-1	100%	96.71%
65.	KY062652.1,Human,RUS,NS16-A97	100%	96.54%
66.	LC129059.1,Human,JPN,HO-61	100%	96.54%
67.	KP342028.1,Human,CHN,SZ94	100%	96.54%
68.	M94155.1,Human,UK,Belm	100%	96.21%
69.	AM118020.1,Human,BCN21	99%	96.04%
70.	AM118018.1,Human,BCN6	99%	96.04%
71.	GU592518.3,Human,RUS,Omsk08-386	99%	95.54%
72.	AF325806.1,Human,Moduganari	100%	94.89%
73.	MK953034.1,Pig,ESP,VC20B	99%	85.01%
74.	MK953029.1,Pig,ESP,C247	99%	84.32%
75.	MK953038.1,Pig,ESP,C429	99%	84.41%
76.	M94157.1,Pig,Cowden	99%	83.94%
77.	M29287.1,Pig,Cowden	99%	83.44%
78.	KX362496.1,Pig,VNM,14249_26	99%	83.11%
79.	KX362485.1,Pig,VNM,14249_25	99%	83.11%
80.	KX362460.1,Pig,VNM,12130_53	99%	83.11%
81.	KT281835.1,Pig,BRA,UEL-576	99%	82.98%
82.	KX453795.1,Pig,IND,UP-101	99%	82.78%
83.	KX374480.1,Pig,IND,UP-100	99%	82.78%
84.	AB889512.1,Pig,CJ10-1	99%	82.78%
85.	MK953028.1,Pig,ESP,P1C	99%	82.70%
86.	KT281832.1,Pig,BRA,UEL-541	95%	83.33%
87.	MT771554.1,Pig,USA,KS_7894	96%	83.25%
88.	MG451700.1,Pig,USA,NC52	95%	83.30%
89.	KT281833.1,Pig,BRA,UEL-554	95%	83.16%

90.	MG451710.1,Pig,USA,MN66	99%	82.42%
91.	AF162434.1,Bovine,USA,WD534tc	99%	82.45%
92.	MG451690.1,Pig,USA,NC38	99%	82.09%
93.	MG451689.1,Pig,USA,NC37	99%	82.09%
94.	MG451659.1,Pig,USA,IL9	99%	82.09%
95.	MG451705.1,Pig,USA,MN57	99%	82.09%
96.	MT771564.1,Pig,USA,MN_1246	99%	81.98%
97.	MK953033.1,Pig,ESP,VC7C	99%	81.88%
98.	MK953030.1,Pig,ESP,VT25C	99%	81.88%
99.	AB889513.1,Pig,CJ13-6	95%	82.47%
100.	KT281829.1,Pig,UEL-217	99%	81.59%
101.	MG451678.1,Pig,USA,NC25	99%	81.43%
102.	MG451724.1,Pig,USA,IA78	99%	81.26%
103.	MG451674.1,Pig,USA,MN21	95%	81.79%
104.	MG451665.1,Pig,USA,WI13	95%	81.44%
105.	KX362507.1,Pig,VNM,14152_5	99%	80.93%
106.	MG451697.1,Pig,USA,IA46-3	99%	80.46%

Supplementary Table S3: BLAST results of VP6 UP-SB21 gene from sloth bear with accession ID, host, country and strain details along with sequence identity and query coverage

**S.No.**	**Accession ID, Host, Country, Strain**	**% coverage**	**% identity**
1.	KX353812.1,Sloth-bear,IND,UP-SB21	100%	100.00%
2.	KX374483.1,Pig,IND,TRI-538	99%	99.63%
3.	KX374481.1,Pig,IND,MIZ-275	100%	99.56%
4.	KX374485.1,Pig,IND,NAG-995	99%	94.27%
5.	KX374495.1,Pig,IND,NAG-993	99%	94.20%
6.	HQ833829.1,Pig,CUK-5	100%	92.36%
7.	KX374482.1,Pig,IND,UP-408	100%	91.62%
8.	AB889518.1,Pig,CJ33-5	100%	91.48%
9.	HQ323753.1,Pig,CUK-6	100%	91.48%

10.	AB889520.1,Pig,CJ59-32	100%	91.40%
11.	KJ814477.1,Pig,KOR,06-52-1	100%	91.33%
12.	AB889502.1,Pig,87-G2	100%	91.03%
13.	AB889501.1,Pig,86-K5	100%	91.03%
14.	AB889514.1,Pig,CJ16-4	100%	90.89%
15.	AB889519.1,Pig,CJ49-4	100%	90.67%
16.	AB889511.1,Pig,CJ3-6	100%	90.66%
17.	LC622317.1,Pig,JPN,Ishi-Im9	99%	90.56%
18.	AB889503.1,Pig,87-I4	100%	90.14%
19.	LC122623.1,Pig,Ishi-1	97%	90.33%
20.	AB889500.1,Pig,86-H5	100%	89.70%
21.	LC622340.1,Pig,JPN,Ishi-Ya1	100%	89.70%
22.	AB889499.1,Pig,86-H3	100%	89.62%
23.	AB889508.1,Pig,93-Z4	100%	89.55%
24.	AB889505.1,Pig,91-G10	100%	89.47%
25.	LC622352.1,Pig,JPN,Ishi-Ya3	99%	89.63%
26.	AB889509.1,Pig,105-4	100%	89.33%
27.	AB889504.1,Pig,91-G7	100%	89.33%
28.	LC622305.1,Pig,JPN,Ishi-Im1	95%	90.12%
29.	LC622374.1,Pig,JPN,HgYa2	98%	89.20%
30.	KX362449.1,Pig,VNM,12129_51	99%	88.51%
31.	LC622328.1,Pig,JPN,Ishi-Ka6	99%	88.49%
32.	M94157.1,Pig,Cowden	100%	88.22%
33.	KX362496.1,Pig,VNM,14249_26	100%	88.07%
34.	LC122612.1,Pig,Tottori-KT01	96%	88.74%
35.	M29287.1,Pig,Cowden	100%	87.86%
36.	KX362460.1,Pig,VNM,12130_53	98%	87.79%
37.	MH282890.1,Pig,USA,OK.5.68	100%	87.11%
38.	AB889516.1,Pig,CJ31-6	100%	86.52%

39.	AB889517.1,Pig,CJ32-3	100%	86.31%
40.	AB889513.1,Pig,CJ13-6	100%	86.22%
41.	KM099256.1,Pig,CZE,P303	95%	87.05%
42.	AF162434.1,Bovine,USA,WD534tc	97%	86.67%
43.	KJ814480.1,Pig,KOR,07-74-11	100%	85.99%
44.	FJ494690.1,,Pig,ROK,06-92-1	100%	85.85%
45.	AB889512.1,Pig,CJ10-1	100%	85.77%
46.	KM099257.1,,Pig,CZE,P21	95%	86.57%
47.	KM099259.1,Pig,CZE,P59	95%	86.56%
48.	KM099258.1,Pig,CZE,P44	95%	86.50%
49.	LC122591.1,Pig,Tochigi-1-1	100%	85.63%
50.	AB889510.1,Pig,134-9	100%	85.48%
51.	KC164674.1,Pig,USA,RV0104	100%	85.49%
52.	KC164677.1,Pig,USA,RV0143	100%	85.42%
53.	AB889507.1,Pig,93-H5	100%	85.19%
54.	KJ814482.1,Pig,KOR,09-15-9	100%	85.11%
55.	KJ814481.1,Pig,KOR,09-15-7	100%	85.11%
56.	FJ494692.1,Pig,06-146-2	100%	85.10%
57.	MN809637.1,Pig,RV0143	98%	85.25%
58.	GQ925781.1,Pig,ROK,CA-2	100%	85.04%
59.	KJ814483.1,Pig,KOR,08-128-1	100%	84.96%
60.	AB889515.1,Pig,CJ27-1	100%	84.73%
61.	HQ185636.1,Human,BS347	100%	84.38%
62.	KX374480.1,Pig,IND,UP-100	99%	84.11%
63.	AM118019.1,Human,BCN9	99%	84.06%
64.	AY786571.1,Human,V966	99%	83.99%
65.	HQ185656.1,Human,YNR001	100%	83.86%
66.	EF528570.1,Human,china,Wu82	100%	83.85%
67.	AY795898.1,Human,V508	99%	83.91%

68.	AY770980.1,Human,v508	99%	83.91%
69.	AF325805.1,Human,Jajeri	99%	83.91%
70.	AB008672.1,Human,JPN,208	100%	83.86%
71.	KY865348.1,Human,CHN,SJZ217	99%	83.84%
72.	KT355391.1,Human,CAU13-1-77	99%	83.84%
73.	KT284781.1,Human,CAU14-1-242	99%	83.84%
74.	KP342039.1,Human,CHN,SZ272	100%	83.79%
75.	KP342028.1,Human,CHN,SZ94	100%	83.79%
76.	KM886901.1Human,Chungnam	99%	83.84%
77.	HQ185667.1,Human,OH567	100%	83.79%
78.	EF641110.1,Human,CMH004,03	99%	83.84%
79.	LC129064.1,Human,JPN,OS-270	99%	83.83%
80.	LC129060.1,Human,JPN,HO-62	99%	83.75%
81.	LC129058.1,Human,JPN,HI-49	99%	83.75%
82.	KT206195.1,Human,ITA,PR713	99%	83.77%
83.	KT206184.1,Human,ITA,PR2593	99%	83.77%
84.	NC_007570.1,Human,UK,Bristol	100%	83.70%
85.	KP776603.1,Human,HUN,ERN6210	99%	83.77%
86.	X59843.2,Human,Bristol,4077-89	100%	83.70%
87.	KX374484.1,Pig,IND,ASM-896	99%	83.73%
88.	LC129065.1,Human,JPN,YA-27	99%	83.81%
89.	HQ896714.1,Human,CAU 10-312	99%	83.69%
90.	LC129061.1,Human,JPN,HO-63	99%	83.72%
91.	KP735972.1,Human,RUS,Omsk08-437	99%	83.69%
92.	GU199224.1,Human,Icheon	100%	83.64%
93.	AM118020.1,Human,BCN21	99%	83.68%
94.	JN934895.2,Human,RUS,Omsk08-436	99%	83.62%
95.	KP735976.1,Human,RUS,Nsk09-B11	99%	83.62%
96.	GU592519.2,Human,RUS,Nsk09-B43	99%	83.62%

97.	M94156.1,Human,Preston	99%	83.61%
98.	LC129063.1,Human,JPN,HO-65	99%	83.62%
99.	GU592517.3,Human,RUS,Nsk08-3414	99%	83.54%
100	AB499614.1,Human,GUP188	99%	83.51%
101	GU592518.3,Human,RUS,Omsk08-386	99%	83.46%
102	KJ814479.1,Pig,KOR,04-155-5	100%	83.43%
103	AM118018.1,Human,BCN6	99%	83.46%
104	LC129059.1,Human,JPN,HO-61	98%	83.62%
105	MN206055.1,Hu,RUS,NS17-A1035	99%	83.32%
106	MN206044.1,Hu,RUS,NS17-A970	99%	83.32%
107	KY062652.1,Human,RUS,NS16-A97	99%	83.32%
108	MN206066.1,Hu,RUS,NS18-A1602	99%	83.17%
109	FJ494691.1,Pig,06-144-2	100%	83.06%
110	AF325806.1,Human,Moduganari	99%	83.10%
111	AB874633.1,Bovine,Y/3/04	100%	82.96%
112	LC622282.1,Bovine,JPN,Ishi-Mi21	99%	82.99%
113	MN809647.1,Pig,RV0104	99%	83.02%
114	LC622293.1,Bovine,JPN,Ishi-Mi39	99%	82.88%
115	AB874631.1,Bovine,Y/1/04	100%	82.77%
116	KJ814478.1,Pig,KOR,04-105-2	100%	82.69%
117	AB874634.1,Bovine,Y/08	100%	82.65%
118	AB108680.1,Bovine,JPN,Yamagata	100%	82.69%
119	M88768.1,Bovine,USA,Shintoku	100%	82.51%
120	AB874630.1,Bovine,Y/03	100%	82.43%
121	LC622271.1,Bovine,JPN,Ishi-Sa	98%	82.61%
122	AB874635.1,Bovine,Y/10	100%	82.28%
123	AB738416.1,Bovine,Toyama	100%	82.13%

Supplementary Table S4: Codon-wise selection pressure shown for VP6 UP-SB37 gene of RCV virus from sloth bear and other hosts

Codon	S	N	dS	dN	Selection Detected
11	7	1	7.591	1.144	Neg. p= 0.041
13	3	0	4.891	0	Neg. p= 0.037
15	3	0	4.891	0	Neg. p= 0.037
16	3	0	6.352	0	Neg. p= 0.022
18	5	0	6.997	0	Neg. p= 0.004
19	3	0	4.19	0	Neg. p= 0.041
21	6	0	7.357	0	Neg. p= 0.003
22	4	0	7.083	0	Neg. p= 0.006
28	4	0	5.536	0	Neg. p= 0.012
29	7	0	9.689	0	Neg. p= 0
30	3	0	4.36	0	Neg. p= 0.032
31	6	0	10.148	0	Neg. p= 0.001
32	7	0	9.689	0	Neg. p= 0
34	9	0	12.457	0	Neg. p= 0
36	4	1	7.384	0.615	Neg. p= 0.016
37	3	1	6.189	0.594	Neg. p= 0.037
38	6	1	8.663	0.678	Neg. p= 0.005
40	4	0	6.388	0	Neg. p= 0.028
41	5	0	6.92	0	Neg. p= 0.004
42	2	0	3.739	0	Neg. p= 0.061
44	9	2	8.193	2.087	Neg. p= 0.053
48	4	0	3.649	0	Neg. p= 0.092
50	7	0	9.689	0	Neg. p= 0
51	2	0	3.683	0	Neg. p= 0.063
52	5	2	7.094	1.367	Neg. p= 0.041
53	2	0	2.606	0	Neg. p= 0.066
54	2	0	2.689	0	Neg. p= 0.061
55	3	0	3	0	Neg. p= 0.037

57	7	0	7	0	Neg. p= 0.001
59	3	0	3	0	Neg. p= 0.04
60	7	0	7	0	Neg. p= 0
61	9	0	10.797	0	Neg. p= 0
62	5	0	5	0	Neg. p= 0.004
63	5	2	6.383	1.477	Neg. p= 0.067
64	5	0	7.208	0	Neg. p= 0.001
65	5	1	6.899	0.454	Neg. p= 0.004
67	7	3	8.822	1.36	Neg. p= 0.005
68	3	0	4.077	0	Neg. p= 0.015
69	8	0	9.623	0	Neg. p= 0.001
70	8	0	5.7	0	Neg. p= 0.002
71	4	0	4	0	Neg. p= 0.012
72	5	0	6.825	0	Neg. p= 0.005
73	14	0	8.357	0	Neg. p= 0.001
74	3	0	3	0	Neg. p= 0.037
75	5	0	5	0	Neg. p= 0.004
76	5	0	5	0	Neg. p= 0.004
77	12	0	7.869	0	Neg. p= 0
78	12	1	8.172	0.747	Neg. p= 0.003
79	3	0	4.079	0	Neg. p= 0.015
80	8	1	5.467	0.772	Neg. p= 0.03
82	3	0	4.083	0	Neg. p= 0.015
83	4	0	5.163	0	Neg. p= 0.004
86	3	0	4.354	0	Neg. p= 0.035
87	7	1	7	0.505	Neg. p= 0.003
88	6	1	6	0.5	Neg. p= 0.007
89	3	0	3.796	0	Neg. p= 0.023
90	6	4	7.458	1.822	Neg. p= 0.028

91	8.5	6.5	9.773	3.051	Neg. p= 0.027
92	5	0	5.257	0	Neg. p= 0.003
93	3	0	4.059	0	Neg. p= 0.015
94	4	1	5.42	0.489	Neg. p= 0.019
95	11	1	7.13	0.812	Neg. p= 0.009
96	8	1	8	0.505	Neg. p= 0.001
97	4	1	4	0.521	Neg. p= 0.05
98	4	0	5.299	0	Neg. p= 0.004
100	3	0	4.254	0	Neg. p= 0.014
101	5	1	5	0.5	Neg. p= 0.018
102	5	0	5	0	Neg. p= 0.004
105	2	0	2.676	0	Neg. p= 0.062
106	4	0	5.762	0	Neg. p= 0.003
107	4	2	5.541	0.878	Neg. p= 0.033
110	4	0	5.772	0	Neg. p= 0.003
111	11	1	11	0.5	Neg. p= 0
113	5	0	6.088	0	Neg. p= 0.002
115	3	0	3	0	Neg. p= 0.039
117	3	0	4.309	0	Neg. p= 0.036
119	7	0	8.798	0	Neg. p= 0.001
120	7	0	7	0	Neg. p= 0
121	4	0	4	0	Neg. p= 0.012
122	7	4	7	2	Neg. p= 0.039
123	5	0	4.964	0	Neg. p= 0.004
124	2	0	5.156	0	Neg. p= 0.017
126	10	0	7.046	0	Neg. p= 0.001
132	7	1	9.424	0.494	Neg. p= 0.001
133	9	0	9	0	Neg. p= 0
136	6	1	8.12	0.442	Neg. p= 0.001

137	6	1	7.614	0.452	Neg. p= 0.002
138	11	1	13.573	0.457	Neg. p= 0
142	4	0	4	0	Neg. p= 0.012
143	4	0	4.169	0	Neg. p= 0.01
144	4	0	5.338	0	Neg. p= 0.005
145	2	0	2.71	0	Neg. p= 0.06
149	5	0	6.556	0	Neg. p= 0.006
150	6	0	6	0	Neg. p= 0.002
151	5	0	6.233	0	Neg. p= 0.002
152	4	0	3.999	0	Neg. p= 0.012
153	7	0	9.366	0	Neg. p= 0
155	4	0	4	0	Neg. p= 0.012
156	9	0	9	0	Neg. p= 0
157	5	0	6.763	0	Neg. p= 0.001
158	12	0	14.767	0	Neg. p= 0
159	12	0	13.946	0	Neg. p= 0
161	2	0	2.723	0	Neg. p= 0.06
162	8	1	8.006	0.5	Neg. p= 0.001
164	11	7	9.967	3.786	Neg. p= 0.035
166	5	0	7.095	0	Neg. p= 0.001
167	2	0	2.711	0	Neg. p= 0.073
168	3	1	3.952	0.496	Neg. p= 0.065
169	3	0	4.056	0	Neg. p= 0.015
170	6	0	6	0	Neg. p= 0.002
171	10	0	12.183	0	Neg. p= 0
172	8	1	8.442	0.487	Neg. p= 0.001
174	7	0	9.505	0	Neg. p= 0.001
175	7	0	6.796	0	Neg. p= 0.001
177	7.5	6.5	9.22	2.976	Neg. p= 0.037

181	4	0	4	0	Neg. p= 0.014
182	8	1	9.881	0.457	Neg. p= 0
183	10	3	10.057	1.496	Neg. p= 0.002
185	6	0	6	0	Neg. p= 0.002
186	14	1	9.005	0.81	Neg. p= 0.002
187	9	0	10.266	0	Neg. p= 0
188	3	0	4.04	0	Neg. p= 0.02
189	10	0	12.191	0	Neg. p= 0
191	5	2	5	1	Neg. p= 0.045
193	6	0	7.905	0	Neg. p= 0
196	5	0	6.624	0	Neg. p= 0.006
197	4	1	4.176	0.49	Neg. p= 0.039
198	5	0	5	0	Neg. p= 0.004
199	5	0	5	0	Neg. p= 0.004
200	3	0	4.071	0	Neg. p= 0.015
201	8	0	10.272	0	Neg. p= 0
202	6	0	6	0	Neg. p= 0.002
203	8	0	8	0	Neg. p= 0
204	9	2	7.388	1.123	Neg. p= 0.007
206	5	1	7.151	0.449	Neg. p= 0.004
207	3	0	4.052	0	Neg. p= 0.015
208	3	0	3	0	Neg. p= 0.037
209	9	1	9.175	0.509	Neg. p= 0
210	6	0	7.741	0	Neg. p= 0
211	8	2	10.026	0.908	Neg. p= 0.001
212	7	0	7	0	Neg. p= 0
213	4	0	5.77	0	Neg. p= 0.012
214	17	0	16.625	0	Neg. p= 0
215	7	0	8.382	0	Neg. p= 0

216	8	4	8.196	1.976	Neg. p= 0.016
217	2	0	2.716	0	Neg. p= 0.072
218	4	0	5.412	0	Neg. p= 0.004
219	5	0	5	0	Neg. p= 0.004
220	3	0	3.877	0	Neg. p= 0.022
221	3	0	2.57	0	Neg. p= 0.059
222	3	0	4.095	0	Neg. p= 0.019
224	5	0	5	0	Neg. p= 0.004
225	3	1	3.571	0.463	Neg. p= 0.069
226	6	0	6	0	Neg. p= 0.001
227	3	1	4.068	0.442	Neg. p= 0.048
228	7	2	7	1	Neg. p= 0.008
229	9	0	5.543	0	Neg. p= 0.007
231	3	0	4.307	0	Neg. p= 0.016
234	7	0	7	0	Neg. p= 0
235	4	0	4	0	Neg. p= 0.012
236	3	0	3	0	Neg. p= 0.037
237	5	1	5.025	0.51	Neg. p= 0.019
238	6	1	7.707	0.45	Neg. p= 0.002
239	8	1	9.376	0.466	Neg. p= 0
240	11	4	13.592	1.826	Neg. p= 0
241	3	1	3.89	0.449	Neg. p= 0.055
243	5	0	5	0	Neg. p= 0.004
244	10	6	11.929	2.776	Neg. p= 0.004
245	11	0	7.148	0	Neg. p= 0.001
247	4	0	5.339	0	Neg. p= 0.004
250	2	0	2.721	0	Neg. p= 0.06
251	11	3	12.725	2.301	Neg. p= 0.004
252	7	1	7	0.5	Neg. p= 0.003

253	3	0	3	0	Neg. p= 0.037
255	6	1	4.649	0.745	Neg. p= 0.056
256	4	2	7.085	1.296	Neg. p= 0.048
259	5.5	1.5	7.629	1.037	Neg. p= 0.026
260	5	0	8.004	0	Neg. p= 0.012
263	2	0	3.752	0	Neg. p= 0.06
266	6	0	10.515	0	Neg. p= 0
267	5	2	6.92	1.384	Neg. p= 0.045
269	3	0	5.848	0	Neg. p= 0.014
270	4	0	7.165	0	Neg. p= 0.004
272	5	0	9.114	0	Neg. p= 0.001
274	3	0	4.152	0	Neg. p= 0.038
275	5	0	9.03	0	Neg. p= 0.006
276	5	0	4.42	0	Neg. p= 0.053
278	4	0	6.868	0	Neg. p= 0.005
281	4	0	6.507	0	Neg. p= 0.009
282	6	0	9.737	0	Neg. p= 0.001
285	4	1	5.818	0.676	Neg. p= 0.038
286	5	0	6.92	0	Neg. p= 0.004
287	3	0	4.152	0	Neg. p= 0.037
288	5	0	6.92	0	Neg. p= 0.004
289	2	0	3.498	0	Neg. p= 0.08
290	3	0	4.152	0	Neg. p= 0.037
291	3	0	5.607	0	Neg. p= 0.015
292	2	0	3.752	0	Neg. p= 0.06
293	6	2	8.304	1.386	Neg. p= 0.02
294	7	0	11.536	0	Neg. p= 0
295	2	0	3.531	0	Neg. p= 0.079
296	3	0	5.928	0	Neg. p= 0.013

297	8	1	11.396	0.682	Neg. p= 0.001
299	3	0	5.687	0	Neg. p= 0.016
304	5	0	7.096	0	Neg. p= 0.004
305	5	0	7.096	0	Neg. p= 0.004
307	5	1	7.158	0.709	Neg. p= 0.017
308	3	0	5.655	0	Neg. p= 0.045
309	6	0	11.17	0	Neg. p= 0
310	3	0	4.258	0	Neg. p= 0.037
312	2	0	3.557	0	Neg. p= 0.086
313	8	3	8.284	2.636	Neg. p= 0.069
314	6	0	10.175	0	Neg. p= 0
318	6	0	8.66	0	Neg. p= 0.001
319	6	0	8.66	0	Neg. p= 0.001
323	3	0	6.118	0	Neg. p= 0.038
326	3	0	5.863	0	Neg. p= 0.02
328	6	1	7.567	0.826	Neg. p= 0.018
329	3	0	4.33	0	Neg. p= 0.037
330	7	0	7.477	0	Neg. p= 0.005
331	2	0	3.911	0	Neg. p= 0.061
333	6	0	6.65	0	Neg. p= 0.009
334	2	0	3.91	0	Neg. p= 0.061
335	3	0	4.33	0	Neg. p= 0.037
336	4	0	5.773	0	Neg. p= 0.012
338	5	0	9.763	0	Neg. p= 0.001
339	4	0	5.773	0	Neg. p= 0.014
340	5	0	7.217	0	Neg. p= 0.004
342	2	0	3.872	0	Neg. p= 0.071
344	5	0	7.217	0	Neg. p= 0.005
345	3	0	5.559	0	Neg. p= 0.018

349	3	0	5.725	0	Neg. p= 0.021
351	5	0	7.921	0	Neg. p= 0.003
352	4	0	5.773	0	Neg. p= 0.012
353	3	0	4.33	0	Neg. p= 0.037
354	3	0	5.358	0	Neg. p= 0.02
355	5.5	2.5	8.075	1.818	Neg. p= 0.052
358	4	0	6.855	0	Neg. p= 0.006
365	4	1	5.773	0.722	Neg. p= 0.045
370	3	0	4.33	0	Neg. p= 0.037
371	2	0	3.921	0	Neg. p= 0.06
375	6	1	11.064	1.03	Neg. p= 0.01
376	3	0	4.33	0	Neg. p= 0.037
377	5	0	4.377	0	Neg. p= 0.063
379	2	0	3.295	0	Neg. p= 0.085
382	4	0	5.773	0	Neg. p= 0.012
385	3	0	6.236	0	Neg. p= 0.013
386	2	0	3.915	0	Neg. p= 0.06
387	4	0	7.529	0	Neg. p= 0.004
392	7	0	6.876	0	Neg. p= 0.012
393	8	0	7.524	0	Neg. p= 0.006
395	3	0	4.33	0	Neg. p= 0.037
396	5	0	7.217	0	Neg. p= 0.004

Supplementary Table S5: Codon-wise selection pressure shown for VP6 UP-SB21 gene of RCV virus from sloth bear and other hosts

Codon	S*	N	dS	dN	Selection detected
1	8	0	8.07	0	Neg. p= 0
2	0	13	0	6.041	Pos. p= 0.015
9	2	0	2.548	0	Neg. p= 0.068
10	15	0	15	0	Neg. p= 0

12	0	13	0	6.03	Pos. p= 0.014
18	1	14	1.046	6.851	Pos. p= 0.025
26	0	8	0	6.694	Pos. p= 0.001
30	0	7	0	3.396	Pos. p= 0.076
32	0	13	0	6.439	Pos. p= 0.015
33	0	9	0	7.123	Pos. p= 0.004
35	8	10	10.169	4.557	Neg. p= 0.075
36	0	5	0	3.765	Pos. p= 0.025
40	0	18	0	8.814	Pos. p= 0.001
41	0	15	0	5.939	Pos. p= 0.076
42	0	19	0	9.048	Pos. p= 0.001
45	0	4	0	3.616	Pos. p= 0.019
49	0	11	0	5.29	Pos. p= 0.018
50	0	18	0	8.616	Pos. p= 0.001
52	0	13	0	6.381	Pos. p= 0.017
54	0	10	0	4.5	Pos. p= 0.05
56	0	19	0	9.542	Pos. p= 0
57	0	9	0	4.331	Pos. p= 0.037
59	2	17	2.198	8.35	Pos. p= 0.039
60	0	7	0	3.383	Pos. p= 0.074
61	15	11	18.371	5.038	Neg. p= 0.001
62	0	9	0	4.448	Pos. p= 0.029
63	0	11	0	8.331	Pos. p= 0.005
64	9	3	9.117	1.49	Neg. p= 0.003
69	0	6	0	2.94	Pos. p= 0.099
71	0	8	0	3.857	Pos. p= 0.053
72	0	7	0	3.604	Pos. p= 0.07
74	0	16	0	13.207	Pos. p= 0
75	5	4	6.226	1.873	Neg. p= 0.069

78	14	7	14.795	3.408	Neg. p= 0.001
79	0	11	0	5.261	Pos. p= 0.019
80	0	9	0	4.101	Pos. p= 0.06
81	1	9	0.733	6.293	Pos. p= 0.013
82	0	10	0	5.176	Pos. p= 0.02
83	0	11	0	9.408	Pos. p= 0
84	0	12	0	5.947	Pos. p= 0.021
90	0	6	0	3.202	Pos. p= 0.089
91	0	14	0	6.488	Pos. p= 0.01
100	0	13	0	6.407	Pos. p= 0.006
103	10	7	11.706	3.292	Neg. p= 0.009
104	0	10	0	4.909	Pos. p= 0.021
107	0	13	0	5.807	Pos. p= 0.022
108	0	8	0	6.083	Pos. p= 0.003
109	0	15	0	7.235	Pos. p= 0.01
110	0	13	0	10.576	Pos. p= 0
111	0	12	0	5.911	Pos. p= 0.009
113	0	16	0	6.835	Pos. p= 0.019
114	0	10	0	4.995	Pos. p= 0.018
117	0	8	0	6.918	Pos. p= 0.001
121	0	10	0	4.793	Pos. p= 0.026
124	0	5	0	3.751	Pos. p= 0.074
125	0	12	0	5.82	Pos. p= 0.011
126	0	12	0	5.998	Pos. p= 0.008
129	1	7	0.969	5.985	Pos. p= 0.051
130	0	12	0	5.513	Pos. p= 0.024
132	0	14	0	6.25	Pos. p= 0.017
133	0	16	0	7.116	Pos. p= 0.01
134	0	14	0	6.653	Pos. p= 0.016

135	0	6	0	2.985	Pos. p= 0.09
136	0	8	0	6.026	Pos. p= 0.016
137	0	16	0	7.876	Pos. p= 0.005
138	0	3	0	2.662	Pos. p= 0.065
141	0	10	0	4.592	Pos. p= 0.052
143	0	8	0	3.627	Pos. p= 0.085
144	0	12	0	4.93	Pos. p= 0.081
146	0	13	0	6.369	Pos. p= 0.008
147	0	13	0	6.238	Pos. p= 0.009
148	0	18	0	8.992	Pos. p= 0.001
150	0.5	14.5	0.569	6.836	Pos. p= 0.023
151	0	13	0	6.256	Pos. p= 0.008
153	0	21	0	12.483	Pos. p= 0
157	9	10	10.608	4.657	Neg. p= 0.061
163	0	12	0	6.001	Pos. p= 0.008
165	0	20	0	9.341	Pos. p= 0.001
166	0	6	0	3	Pos. p= 0.088
168	0	7	0	5.702	Pos. p= 0.003
169	0	5	0	4.115	Pos. p= 0.058
170	0	9	0	4.311	Pos. p= 0.038
171	0	11	0	9.268	Pos. p= 0
173	0	9	0	6.362	Pos. p= 0.012
178	0	11	0	5.332	Pos. p= 0.016
179	1	21	0.986	14.828	Pos. p= 0
182	0	14	0	6.955	Pos. p= 0.004
183	0	15	0	7.186	Pos. p= 0.005
184	0	21	0	10.328	Pos. p= 0
185	1	10	0.879	5.78	Pos. p= 0.032
186	0	11	0	5.001	Pos. p= 0.033

189	0	10	0	4.591	Pos. p= 0.048
191	0	10	0	4.867	Pos. p= 0.048
193	0	10	0	7.122	Pos. p= 0.013
194	0	11	0	5.021	Pos. p= 0.035
195	0	7	0	6.189	Pos. p= 0.001
199	0	12	0	6.341	Pos. p= 0.008
200	0	7	0	6.248	Pos. p= 0.001
201	0	10	0	5	Pos. p= 0.017
202	0	8	0	3.999	Pos. p= 0.039
205	0	20	0	8.565	Pos. p= 0.007
207	4	10	3.067	7.974	Pos. p= 0.078
208	10	11	11.913	5.109	Neg. p= 0.044
209	0	10	0	4.998	Pos. p= 0.023
210	0	4	0	3.637	Pos. p= 0.02
211	0	8	0	3.981	Pos. p= 0.041
213	1	10	1.014	4.965	Pos. p= 0.08
214	0	10	0	4.88	Pos. p= 0.044
216	5	1	5	0.5	Neg. p= 0.018
217	0	12	0	5.908	Pos. p= 0.022
218	0	15	0	8.664	Pos. p= 0.001
220	1	7	1.012	5.803	Pos. p= 0.063
221	0	18	0	8.973	Pos. p= 0.001
225	0	12	0	5.667	Pos. p= 0.015
226	1	14	0.726	9.906	Pos. p= 0.001
227	0	21	0	15.667	Pos. p= 0
228	0	9	0	4.018	Pos. p= 0.072
229	0	11	0	4.975	Pos. p= 0.035
230	0	13	0	5.218	Pos. p= 0.091
233	0	13	0	6.456	Pos. p= 0.006

234	11	11	13.664	5.011	Neg. p= 0.017
237	0	21	0	9.107	Pos. p= 0.004
239	3	1	3.42	0.485	Neg. p= 0.083
240	0	9	0	7.031	Pos. p= 0
242	0	14	0	6.422	Pos. p= 0.011
243	0	5	0	4.336	Pos. p= 0.012
245	0	11	0	5.106	Pos. p= 0.026
250	0	6	0	4.904	Pos. p= 0.033
251	0	10	0	8.198	Pos. p= 0
252	0	8	0	3.956	Pos. p= 0.043
253	0	8	0	3.572	Pos. p= 0.097
255	0	12	0	5.806	Pos. p= 0.012
256	0	13	0	6.392	Pos. p= 0.013
258	0	19	0	8.986	Pos. p= 0.003
259	0	10	0	4.815	Pos. p= 0.025
260	1	11	1.044	5.662	Pos. p= 0.056
261	0	8	0	4.332	Pos. p= 0.035
263	0	10	0	4.902	Pos. p= 0.021
264	0	13	0	6.27	Pos. p= 0.008
267	0	9	0	4.466	Pos. p= 0.028
268	0	9	0	4.759	Pos. p= 0.026
269	1	6	0.539	5.319	Pos. p= 0.014
270	0	10	0	4.515	Pos. p= 0.048
274	8	3	9.641	1.486	Neg. p= 0.003
276	2	7	1.191	5.801	Pos. p= 0.033
277	1	11	1.064	8.064	Pos. p= 0.017
278	0	8	0	6.761	Pos. p= 0.009
279	0	9	0	4.018	Pos. p= 0.072
280	0	23	0	16.553	Pos. p= 0

282	0	6	0	3	Pos. p= 0.088
283	0	7	0	3.434	Pos. p= 0.067
287	0	13	0	6.281	Pos. p= 0.008
288	1	11	1.035	5.408	Pos. p= 0.063
289	0	8	0	5.958	Pos. p= 0.029
290	0	11	0	8.05	Pos. p= 0.003
292	1	9	0.734	6.478	Pos. p= 0.012
294	0	8	0	3.981	Pos. p= 0.046
295	0	14	0	6.895	Pos. p= 0.004
297	0	9	0	7.368	Pos. p= 0.002
300	0	9	0	7.858	Pos. p= 0
302	0	14	0	11.315	Pos. p= 0
303	0	8	0	3.713	Pos. p= 0.087
304	0	9	0	4.372	Pos. p= 0.034
305	0	8	0	3.83	Pos. p= 0.055
307	0	14	0	6.526	Pos. p= 0.009
312	0	15	0	7.288	Pos. p= 0.01
313	12	9	12.726	4.375	Neg. p= 0.013
314	0	14	0	6.901	Pos. p= 0.011
318	0	9	0	7.51	Pos. p= 0.001
320	0	8	0	4.237	Pos. p= 0.039
321	0	9	0	4.793	Pos. p= 0.026
323	12	5	12.374	2.463	Neg. p= 0.001
326	10	11	11.487	5.384	Neg. p= 0.066
329	0	11	0	6.249	Pos. p= 0.003
335	3	17	3	8.5	Pos. p= 0.06
339	2	17	2.06	8.387	Pos. p= 0.029
345	0	8	0	3.998	Pos. p= 0.05
346	2	15	2.169	7.282	Pos. p= 0.067

356	5	1	5.026	0.499	Neg. p= 0.017

* N = non-synonymous and S= synonymous substitutions; dN and dS =their respective rates

Supplementary Table S6: BLAST results of VP7 UP-SB37 gene from sloth bear with accession ID, host, country and strain details along with sequence identity and query coverage

S. No.	Accession ID, Host, Country, Strain	% coverage	% identity
1.	KX442661.1,Sloth-bear,IND,UP-SB37	100%	100.00%
2.	AB905210.1,Pig,CJ10-1	97%	88.40%
3.	LC122610.1,Pig,Tottori-KT01	97%	87.14%
4.	KJ814499.1,Pig,KOR,09-15-7	96%	86.66%
5.	EF464655.1,Pig,134,04-2	96%	86.47%
6.	AB905223.1,Pig,87-I4	97%	86.16%
7.	KJ814497.1,Pig,KOR,07-60-4	96%	86.00%
8.	AB905213.1,Pig,CJ27-1	97%	85.97%
9.	AB905229.1,Pig,105-4	97%	85.97%
10.	MN809638.1,Pig,RV0143	97%	85.96%
11.	AB905222.1,Pig,87-G2	97%	85.69%
12.	EF464657.1,Pig,43,06-22	96%	85.62%
13.	EF464656.1,Pig,43,06-16	96%	85.62%
14.	KJ814500.1,Pig,KOR,61-12	96%	85.61%
15.	LC122600.1,Pig,Tochigi-2	97%	85.57%
16.	AB905219.1,Pig,86-H3	97%	85.53%
17.	AB905215.1,Pig,CJ32-3	97%	85.50%
18.	AB905220.1,Pig,86-H5	97%	85.43%
19.	KJ814498.1,Pig,KOR,07-109-12	96%	85.32%
20.	KJ814496.1,Pig,KOR,06-281-4	96%	85.23%
21.	KJ814495.1,Pig,KOR,06-176-1	96%	85.23%
22.	EF464654.1,Pig,344-04-7	96%	84.86%
23.	KM099270.1,Pig,CZE,P44	96%	84.81%

24.	AB905230.1,Pig,134-9	97%	83.66%
25.	AB905209.1,Pig,CJ3-6	97%	83.30%
26.	KX353813.1,Sloth-bear,IND,UP-SB19	96%	79.98%
27.	KX825945.1,Pig,IND,NAG-995	96%	79.04%
28.	LC122621.1,Pig,Ishi-1	97%	79.00%
29.	KJ814506.1,Pig,KOR,11-58-4	96%	78.40%
30.	KJ814508.1,Pig,KOR,1027	96%	78.31%
31.	KJ814503.1,Pig,KOR,06-69-1	96%	78.14%
32.	KJ814507.1,Pig,KOR,11-58-7,2011	96%	78.12%
33.	M61101.1,Pig,UK,Cowden	97%	78.06%
34.	KJ814502.1,Pig,KOR,06-52-2	96%	78.05%
35.	AF225554.1,Human,T97,190	97%	77.98%
36.	KP735970.1,Human,RUS,Omsk08-437	97%	77.94%
37.	AF225555.1,Human,T97,194	97%	77.94%
38.	X77258.1,Human,UK,Preston	97%	77.90%
39.	AM118023.1,Human,BCN21	97%	77.89%
40.	AF225552.1,Human,T97,126	97%	77.88%
41.	KJ814505.1,Pig,KOR,09-84-5	96%	77.85%
42.	KT284784.1,Human,CAU14-1-242	97%	77.83%
43.	JQ177070.2,Human,RUS,Nsk08-3414	97%	77.83%
44.	KP735974.1,Human,RUS,Nsk09-B11	97%	77.83%
45.	JN934901.1,Human,RUS,Nsk09-B43	97%	77.83%
46.	U20989.1,Human,BF	97%	77.81%
47.	MZ516919.1,Human,USA,BUDS0068	97%	77.81%
48.	AF225561.1,Human,KA4,949	97%	77.79%
49.	AF225558.1,Human,T97,330	97%	77.79%

Supplementary Table S7: Codon-wise selection pressure shown for VP7 gene of RCV virus from sloth bear (UP-SB37 and UP-SB19) and other hosts

Codon	Partition	S	N	dS	dN	Selection detected
17	1	0	8	0	3.794	Pos. p= 0.059
23	1	0	13	0	5.68	Pos. p= 0.03
27	1	0	8	0	3.995	Pos. p= 0.039
29	1	0	9	0	4.072	Pos. p= 0.076
32	1	0	10	0	4.914	Pos. p= 0.021
33	1	0	15	0	7.405	Pos. p= 0.003
34	1	0	8	0	3.866	Pos. p= 0.051
42	1	1	12	1.096	5.962	Pos. p= 0.053
45	1	0	8	0	3.861	Pos. p= 0.094
46	1	1	18	1.121	8.658	Pos. p= 0.01
47	1	0	9	0	4.049	Pos. p= 0.082
48	1	0	14	0	6.788	Pos. p= 0.015
53	1	1	14	1.039	6.876	Pos. p= 0.024
54	1	0	10	0	6.157	Pos. p= 0.034
56	1	0	10	0	4.89	Pos. p= 0.022
63	1	0	9	0	4.445	Pos. p= 0.067
66	1	0	15	0	6.583	Pos. p= 0.017
68	1	0	8	0	3.818	Pos. p= 0.062
72	1	0	6	0	2.972	Pos. p= 0.093
73	1	0	8	0	3.825	Pos. p= 0.056
79	1	0	7	0	3.476	Pos. p= 0.073
86	1	0	6	0	2.999	Pos. p= 0.088
89	1	0	16	0	7.844	Pos. p= 0.002
90	1	6	0	3.989	0	Neg. p= 0.026
91	1	0	7	0	3.469	Pos. p= 0.077
94	1	0	13	0	6.329	Pos. p= 0.009

95	1	0	9	0	4.354	Pos. p= 0.039
96	1	0	12	0	5.637	Pos. p= 0.016
97	1	0	8	0	3.751	Pos. p= 0.065
101	1	0	8	0	3.906	Pos. p= 0.047
108	1	0	11	0	5.247	Pos. p= 0.019
113	1	0	7	0	3.353	Pos. p= 0.08
117	1	3	1	4.16	0.485	Neg. p= 0.056
125	1	0	9	0	4.079	Pos. p= 0.071
128	1	0	11	0	5.066	Pos. p= 0.045
133	1	0	10	0	4.582	Pos. p= 0.061
139	1	0	9	0	4.26	Pos. p= 0.043
142	1	4	2	4.111	0.995	Neg. p= 0.093
148	1	0	10	0	4.515	Pos. p= 0.063
154	1	0	9	0	4.194	Pos. p= 0.049
157	1	0	7	0	3.371	Pos. p= 0.084
161	1	0	11	0	5.325	Pos. p= 0.039
165	1	0	8	0	4	Pos. p= 0.039
166	1	0	7	0	3.413	Pos. p= 0.07
169	1	0	10	0	4.776	Pos. p= 0.031
181	1	0	8	0	3.819	Pos. p= 0.056
182	1	0	10	0	4.901	Pos. p= 0.022
185	1	0	8	0	4.208	Pos. p= 0.034
186	1	1	10	1.012	4.97	Pos. p= 0.079
187	1	0	11	0	5.303	Pos. p= 0.019
188	1	0	7	0	3.462	Pos. p= 0.063
189	1	0	8	0	3.781	Pos. p= 0.098
191	1	0	9	0	4.269	Pos. p= 0.049
192	1	0	11	0	5.358	Pos. p= 0.028
193	1	0	9	0	4.418	Pos. p= 0.031

195	1	0	9	0	4.648	Pos. p= 0.024
199	1	0	7	0	4.387	Pos. p= 0.088
203	1	1.5	13.5	1.599	6.688	Pos. p= 0.063
205	1	0	8	0	3.855	Pos. p= 0.052
207	1	6	0	3.654	0	Neg. p= 0.042
208	1	0	14	0	6.905	Pos. p= 0.015
210	1	0	13	0	6.331	Pos. p= 0.007
211	1	0	10	0	4.904	Pos. p= 0.021
216	1	0	8	0	3.849	Pos. p= 0.097
218	1	0	12	0	5.265	Pos. p= 0.037
220	1	0	13	0	6.217	Pos. p= 0.011
225	1	0	7	0	3.423	Pos. p= 0.068
226	1	0	9	0	4.466	Pos. p= 0.028
233	1	0	9	0	4.259	Pos. p= 0.045
245	1	0	8	0	3.942	Pos. p= 0.058
246	1	0	10	0	4.52	Pos. p= 0.063
248	1	4	0	2.759	0	Neg. p= 0.079
250	1	0	8	0	3.836	Pos. p= 0.062
251	1	0	14	0	6.507	Pos. p= 0.01
252	1	0	9	0	4.028	Pos. p= 0.082
260	1	0	14	0	6.702	Pos. p= 0.017
271	1	2	0	2.312	0	Neg. p= 0.083
272	1	0	7	0	3.713	Pos. p= 0.064
273	1	3	0	3.072	0	Neg. p= 0.034

Supplementary Table S8: BLAST results of NSP4 gene from sloth bear with accession ID, host, country and strain details along with sequence identity and query coverage

**S.No.**	**Accession ID, Host, Country, Strain**	**% coverage**	**%identity**
1.	KX442662.1,SB,IND,UP-SB37	100%	100.00%
2.	KY783644.1,Pig,IND,Por-993	100%	98.95%

3.	KY909976.1,Pig,CAN,A10-122	100%	88.33%
4.	KY909975.1,Pig,CAN,A10-108	100%	88.33%
5.	KY909972.1,Pig,CAN,A5-36	100%	87.87%
6.	KY783647.1,Pig,IND,KL-224	100%	87.21%
7.	KY909973.1,Pig,CAN,A8-158	100%	87.11%
8.	AF093202.1,Pig,UAS,Cowden-virulent	100%	86.46%
9.	MH282894.1,Pig,USA,OK.5.68	100%	86.25%
10.	KX373856.1,Pig,UEL-57	100%	86.16%
11.	MZ218321.1,Pig,366R-k141_226904	100%	86.16%
12.	LC622311.1,Pig,JPN,Ishi-Im1	100%	85.95%
13.	LC122608.1,Pig,Tochigi-2	100%	85.80%
14.	LC622333.1,Pig,JPN,Ishi-Ka6	100%	85.74%
15.	AF093203.1,Pig,USA,Cowden-attenuated	100%	85.83%
16.	LC122630.1,Pig,Ishi-1	100%	85.12%
17.	LC122619.1,Pig,Tottori-KT01	100%	85.12%
18.	KX373857.1,Pig,UEL-77	100%	84.91%
19.	KX373863.1,Pig,UEL-1436	100%	84.70%
20.	KM099249.1,Pig,CZE,P9	99%	84.81%
21.	KC164673.1,Pig,USA,RV0104	98%	84.96%
22.	KX373855.1,Pig,UEL-33	100%	84.49%
23.	MN809642.1,Pig,RV0143	100%	84.28%
24.	LC622322.1,Pig,JPN,Ishi-Im9	100%	84.38%
25.	KY783643.1,Pig,IND,Por-103	100%	84.10%
26.	KY783645.1,Pig,IND,ASM-132	100%	84.10%
27.	KX373858.1,Pig,UEL-154	100%	84.07%
28.	KC164676.1,Pig,USA,RV0143	100%	84.07%
29.	KY783642.1,Pig,IND,Por-100	100%	83.89%
30.	KY783641.1,Pig,IND,Por-98	100%	83.89%
31.	KY783649.1,Pig,IND,UP-404	100%	83.89%

32.	KX373859.1,Pig,UEL-203	100%	83.99%
33.	LC622368.1,Pig,JPN,HgYa1	100%	83.89%
34.	KY783646.1,Pig,IND,ASM-140	100%	83.68%
35.	KM099248.1,Pig,CZE,P8	97%	84.12%
36.	LC122598.1,Pig,Tochigi-1-1	100%	83.44%
37.	KX373861.1,Pig,UEL-576	100%	83.37%
38.	MN395882.1, Human,BRA,SD137	99%	83.12%
39.	KM099250.1,Pig,CZE,P303	98%	83.37%
40.	MN395899.1,Human,BRA,BP209	99%	82.91%
41.	MN395900.1, Human,BRA,BP213	99%	82.91%
42.	MN395883.1, Human,BRA,SD181	99%	82.91%
43.	KX373860.1,Pig,UEL-369	100%	82.81%
44.	KY783648.1,Pig,IND,TRI-542	100%	82.64%
45.	KX373862.1,Pig,UEL-916	100%	82.46%
46.	OK491938.1,Pig,IND,NIV94306	95%	83.11%
47.	OK491937.1,Pig,IND,NIV94294	95%	83.11%
48.	OK491936.1,Pig,IND,NIV94319	95%	83.08%
49.	OK491935.1,Pig,IND,NIV94285	95%	83.08%
50.	OK491934.1,Pig,IND,NIV94292	95%	83.08%
51.	OK491933.1,Pig,IND,NIV94291	95%	83.08%
52.	OK491932.1,Pig,IND,NIV94289	95%	83.08%
53.	LC622357.1,Pig,JPN,Ishi-Ya3	98%	82.59%
54.	LC622346.1,Pig,JPN,Ishi-Ya1	98%	82.59%
55.	KM099252.1,Pig,CZE,P44	100%	82.19%
56.	KM099251.1,Pig,CZE,P21	100%	82.16%
57.	KX373864.1,Pig,UEL-1515	100%	81.84%
58.	KM099253.1,Pig,CZE,P59	98%	81.97%
59.	KY909979.1,Pig,CAN,N-A12-26	100%	80.75%

Supplementary Table S9: Codon-wise selection pressure shown for NSP4 gene of RCV virus from sloth bear (UP-SB37) and other hosts

Codon	S*	N	dS	dN	Selection detected
8	10	0	12.569	0	Neg. p= 0
13	4	1	4.012	0.505	Neg. p= 0.046
14	7	2	8.813	0.907	Neg. p= 0.002
18	5.5	2.5	5.509	1.251	Neg. p= 0.054
21	6	2	7.539	0.908	Neg. p= 0.006
22	6	2	6.025	0.998	Neg. p= 0.019
24	4	1	4	0.5	Neg. p= 0.045
25	5	1	6.335	0.5	Neg. p= 0.008
26	1	12	1.03	5.913	Pos. p= 0.045
27	7	1	4.028	0.833	Neg. p= 0.098
28	3	0	3	0	Neg. p= 0.039
30	0	9	0	4.435	Pos. p= 0.03
33	6	0	3.343	0	Neg. p= 0.046
34	7	0	7	0	Neg. p= 0
35	7	0	8.89	0	Neg. p= 0
37	3	0	3	0	Neg. p= 0.045
40	14	1	8.587	0.828	Neg. p= 0.003
41	5	4	6.038	1.921	Neg. p= 0.081
44	3	0	3.807	0	Neg. p= 0.018
49	5	0	2.79	0	Neg. p= 0.077
51	7	5	7.469	2.534	Neg. p= 0.056
54	8	4	8.382	1.955	Neg. p= 0.014
57	6	0	6	0	Neg. p= 0.002
60	7.5	8.5	10.376	3.737	Neg. p= 0.045
63	4	0	4.939	0	Neg. p= 0.006
64	8	1	10.06	0.455	Neg. p= 0
65	5	3	5.48	1.498	Neg. p= 0.069

67	6	0	7.271	0	Neg. p= 0
68	6	6	7.582	2.717	Neg. p= 0.069
69	6	6	7.57	2.718	Neg. p= 0.069
76	5	2	6.349	0.904	Neg. p= 0.016
78	5	0	6.35	0	Neg. p= 0.001
79	6	2	7.285	0.956	Neg. p= 0.008
80	8	0	9.695	0	Neg. p= 0
82	4	0	4.94	0	Neg. p= 0.006
86	5	3	5.941	1.46	Neg. p= 0.051
87	6	0	7.364	0	Neg. p= 0.003
90	8	0	9.613	0	Neg. p= 0
94	3	0	3.812	0	Neg. p= 0.018
96	5	0	5	0	Neg. p= 0.004
98	11	0	6.817	0	Neg. p= 0.002
99	3	0	3.802	0	Neg. p= 0.018
101	7	6	8.853	2.716	Neg. p= 0.032
105	6	0	7.6	0	Neg. p= 0
106	7	0	8.834	0	Neg. p= 0
110	10	1	10.43	0.502	Neg. p= 0
113	6	0	7.318	0	Neg. p= 0
114	8	1	8	0.551	Neg. p= 0.002
115	5	2	5.497	0.957	Neg. p= 0.03
116	9	1	8.944	0.509	Neg. p= 0
117	2	0	2.177	0	Neg. p= 0.099
118	4	3	4.916	1.408	Neg. p= 0.097
120	12	0	13.777	0	Neg. p= 0
125	10.167	9.833	13.334	4.431	Neg. p= 0.014
126	6	2	7.601	0.905	Neg. p= 0.006
131	7	7	8.14	3.348	Neg. p= 0.081

132	0.833	13.167	0.858	6.499	Pos. p= 0.027
133	6	5	6.848	2.452	Neg. p= 0.079
140	7	0	8.876	0	Neg. p= 0
142	9	0	11.422	0	Neg. p= 0
143	7	1	8.854	0.453	Neg. p= 0.001
144	10	2	10.049	0.998	Neg. p= 0.001
145	3	0	3	0	Neg. p= 0.038
147	8	0	4.94	0	Neg. p= 0.012
149	5	0	5	0	Neg. p= 0.004
150	2	0	2.474	0	Neg. p= 0.075

* N = non-synonymous and S= synonymous substitutions; dN and dS =their respective rates
